# Empowering Health Care Education Through Learning Analytics: In-depth Scoping Review

**DOI:** 10.2196/41671

**Published:** 2023-05-17

**Authors:** Iva Bojic, Maleyka Mammadova, Chin-Siang Ang, Wei Lung Teo, Cristina Diordieva, Anita Pienkowska, Dragan Gašević, Josip Car

**Affiliations:** 1 Lee Kong Chian School of Medicine Nanyang Technological University Singapore Singapore; 2 Department of Human Centred Computing Faculty of Information Technology Monash University Melbourne Australia

**Keywords:** distance education and web-based learning, distributed learning environments, data science applications in education, 21st century abilities, cooperative and collaborative learning, COVID-19, education, digital, data, student

## Abstract

**Background:**

Digital education has expanded since the COVID-19 pandemic began. A substantial amount of recent data on how students learn has become available for learning analytics (LA). LA denotes the “measurement, collection, analysis, and reporting of data about learners and their contexts, for purposes of understanding and optimizing learning and the environments in which it occurs.”

**Objective:**

This scoping review aimed to examine the use of LA in health care professions education and propose a framework for the LA life cycle.

**Methods:**

We performed a comprehensive literature search of 10 databases: MEDLINE, Embase, Web of Science, ERIC, Cochrane Library, PsycINFO, CINAHL, ICTP, Scopus, and IEEE Explore. In total, 6 reviewers worked in pairs and performed title, abstract, and full-text screening. We resolved disagreements on study selection by consensus and discussion with other reviewers. We included papers if they met the following criteria: papers on health care professions education, papers on digital education, and papers that collected LA data from any type of digital education platform.

**Results:**

We retrieved 1238 papers, of which 65 met the inclusion criteria. From those papers, we extracted some typical characteristics of the LA process and proposed a framework for the LA life cycle, including digital education content creation, data collection, data analytics, and the purposes of LA. Assignment materials were the most popular type of digital education content (47/65, 72%), whereas the most commonly collected data types were the number of connections to the learning materials (53/65, 82%). Descriptive statistics was mostly used in data analytics in 89% (58/65) of studies. Finally, among the purposes for LA, understanding learners’ interactions with the digital education platform was cited most often in 86% (56/65) of papers and understanding the relationship between interactions and student performance was cited in 63% (41/65) of papers. Far less common were the purposes of optimizing learning: the provision of at-risk intervention, feedback, and adaptive learning was found in 11, 5, and 3 papers, respectively.

**Conclusions:**

We identified gaps for each of the 4 components of the LA life cycle, with the lack of an iterative approach while designing courses for health care professions being the most prevalent. We identified only 1 instance in which the authors used knowledge from a previous course to improve the next course. Only 2 studies reported that LA was used to detect at-risk students during the course’s run, compared with the overwhelming majority of other studies in which data analysis was performed only after the course was completed.

## Introduction

### Background

Health care education includes preregistration education, postregistration education, and lifelong learning for practicing clinicians [1]. The goal of health care professions education is to improve the potential of people—doctors, nurses, and allied health care professionals—to meet the health needs of patients, communities, and populations [2]. This requires ensuring that students learn what they need to know and be able to do, assessing the extent to which educational goals and outcomes are achieved and monitoring the quality of educational programs [1].

Digital education can be defined as learning through the use of electronic technology. It is becoming increasingly popular in academic and clinical settings [3] for continuing education [4]. Although education has evolved from an offline to a web-based environment through internet access, the COVID-19 pandemic has made web-based learning the standard delivery method worldwide [5,6]. Thus, the *new normal* educational practices based on technology-enabled globalization, borderless teaching, and seamless learning are emerging [7]. The transition from an in-person learning format to a digital learning format brings new challenges [8] but also opens new opportunities for educators through the use of a digital education platform. Digital education platforms are web-based systems that automate the administration of learning activities, including delivery, tracking, and reporting activities [9]. They subsequently generate substantial amounts of data on student learning, which are available for further analysis with learning analytics (LA).

LA can be defined as the “measurement, collection, analysis, and reporting of data about learners and their contexts, for purposes of understanding and optimizing learning and the environments in which it occurs” [10]. LA refers to the collection and analysis of data from digital education platforms. Data analysis is used to determine student engagement in various digital learning activities to improve student learning. Specifically, LA differs from traditional educational analyses in 3 ways. First, data sets are typically much larger because of the strong quantitative focus, allowing for a higher degree of confidence in the generalizability of the results [11]. Second, data collected through technology has a finer granularity of variables than those collected via self-report, interview, or observational data [12]. Finally, the data are usually longitudinal [11], meaning that the methods and processes used to collect the data can incorporate a temporal dimension into the analysis [13]. These features provide opportunities for new insights by allowing learners to record, analyze, and retrieve their learning processes and outcomes for future use [14].

Given that LA provides powerful mechanisms for understanding, interpreting, and shaping learning processes and experiences using data-driven methods, research in this field has gained popularity since its inception as a scientific discipline in 2011 [15]. The number of studies using the keyword *learning analytics* in SciVerse Scopus between 2011 and 2021 was initially in the 2-digit range (n=29) but has since steadily increased, peaking at 917 in 2019. Despite a minor decline in the past 2 years (n=911 in 2020 and n=825 in 2021), the area continues to generate sizable interest.

### Literature Gaps and Research Questions

Although many studies have been conducted in the last decade that demonstrate the usefulness of LA for health care professions education, we have been able to identify only 2 reviews on this topic [14,16]. Their limitations were that both covered literature reviews only up to 2017, and one of the reviews included data from a single database (ie, MEDLINE). Moreover, they did not propose any framework to analyze the reviewed literature. On the basis of our literature review, which included papers from 10 databases, we identified 4 components of LA. Those 4 components were conceptualized in the form of a life cycle. The life cycle is a cyclical process that explains, in 4 stages, what digital learning content is created, what data are gathered from the content, and how the data are analyzed and used to achieve learning purposes. The first stage of the life cycle involves the creation of learning content. The content could be an assessment or some other type of digital material. The gathering of data occurs in the second stage. It entails gathering data from various sources, such as learners’ interactions with digital learning content and their performance on assessments and tests. This can be as simple as mouse clicks or key presses or as complex as time spent on different pages or interaction with videos or audio files. The interaction with the material generates data that can be analyzed using analytics tools in the third stage of the life cycle. Finally, in the fourth stage of the life cycle, the data analysis results would be used to help improve learner performance, the quality of learning experience, or both.

The idea of an LA life cycle is not new and was previously discussed in a study by Clow [17]. The study proposed a 4-step LA model that includes learners, data, metrics, and interventions. On the basis of the model, learners are people who learn, data are what we collect about learner behavior, metrics are numbers or statistics that describe data, and interventions are specific programs or sets of steps to provide learners with support in an area of need. Although our proposed life cycle model shared some similarities with Clow’s model, the main difference between the 2 models is that the first stage of our LA life cycle is referred to as *content* and not *learner*. We choose to exclude *learner* from our life cycle because previous research has shown that LA is highly content dependent [18]. When designing a course, it is critical to spell out content curation (the totality of what is to be taught in a course). It forms the basis for teaching by determining which topics, concepts, facts, skills, and values are expected to be taught and learned. With a deep dive into what goes into teaching and learning, we can make an informed decision about what variables and metrics can be tracked and analyzed to reveal learners’ preferences and engagement. In other words, content has implications for what data are collected, how such data need to be preprocessed, what approaches can be applied to analyze data, and what can be done to optimize learning outcomes. The research questions that guided this review were as follows:

1. What types of content are used in LA studies in health care professions education?
2. What types of data are collected in health care professions education through content engagement?
3. What approaches are used for data analytics in health care professions education?
4. What are the purposes of analyzing the collected data in health care professions education?

## Methods

To provide a comprehensive overview of the use of LA in health care professions education, we analyzed the literature by conducting a scoping review. This study used the PRISMA-ScR (Preferred Reporting Items for Systematic Reviews and Meta-Analyses extension for Scoping Reviews) standards [19].

### Data Sources and Searches

We searched the following databases for papers written in English from January 2010 to October 2021: MEDLINE, Embase, Web of Science, ERIC, Cochrane Library, PsycINFO, CINAHL, ICTP, Scopus, and IEEE Explore. The year 2010 was used as the starting point for our search, as the field of LA was formally recognized after the first International Conference on LA and Knowledge in 2011 [20]. We developed an initial search strategy based on a past publication on digital health education [21]. After initial title and abstract screening, we used the Search Refiner and Word Frequency Analyzer tools [22] to refine our search strategy further. Finally, we incorporated terms denoting different digital education platforms seen in the initially screened literature (Table S1 in Multimedia Appendix 1). Our final search strategy comprised (1) terms denoting health care professionals, (2) terms denoting different web-based learning environments, and (3) terms relating to LA. Multimedia Appendix 2 contains the final search strategies for all databases.

### Study Selection

We included peer-reviewed publications that met the following criteria: papers on health care professions education, papers on digital education, and papers that collected LA data. We excluded papers that collected data about patient education; educators and not learners; and multimodal LA, abstracts, presentations, reviews, and commentary columns and papers where full texts could not be obtained (Textbox 1).

In total, 2 reviewers (IB and MM) performed title, abstract, and full-text screening of all publications retrieved using the first version of our search strategy. After the first round of screening, they defined a detailed set of inclusion and exclusion criteria and clearly explained the defined criteria to the other 4 reviewers (AP, CD, WLT, and CSA). All 6 reviewers worked in pairs sequentially and evaluated all retrieved papers using our final search strategy. We resolved disagreements on study selection by consensus and discussion with other reviewers.

**Inclusion and exclusion criteria for study selection.**

**Inclusion criteria**

- Articles about health professions education
- Articles about digital learning
- Collected digital learning analytics data from a learning management system

**Exclusion criteria**

- Articles about patient education
- Collected data about educators and not learners
- Collected multimodal learning analytics data
- Reviews and commentary columns

### Data Extraction and Synthesis

A total of 3 reviewers (IB, MM, and CSA) collaborated on a data charting form to select the variables to be extracted. In an iterative process, the 3 reviewers separately charted the data, reviewed the findings, and modified the data extraction form. The 3 reviewers then grouped study data by type of digital education content, type of data obtained from student interactions with digital education platforms, analytics approaches used to process the collected data, and finally the purposes of the LA. We resolved disagreements on data extraction by consensus and discussion with other reviewers. Upon the completion of data extraction, the data were collated, summarized, and subsequently organized into groups according to similarities and differences. Tentative codes were added to these groups and iteratively discussed and revised to generate a comprehensive map of terms. The map was then used to conduct a narrative synthesis, which allowed for the identification of key themes and patterns within the data.

## Results

**Figure 1:**
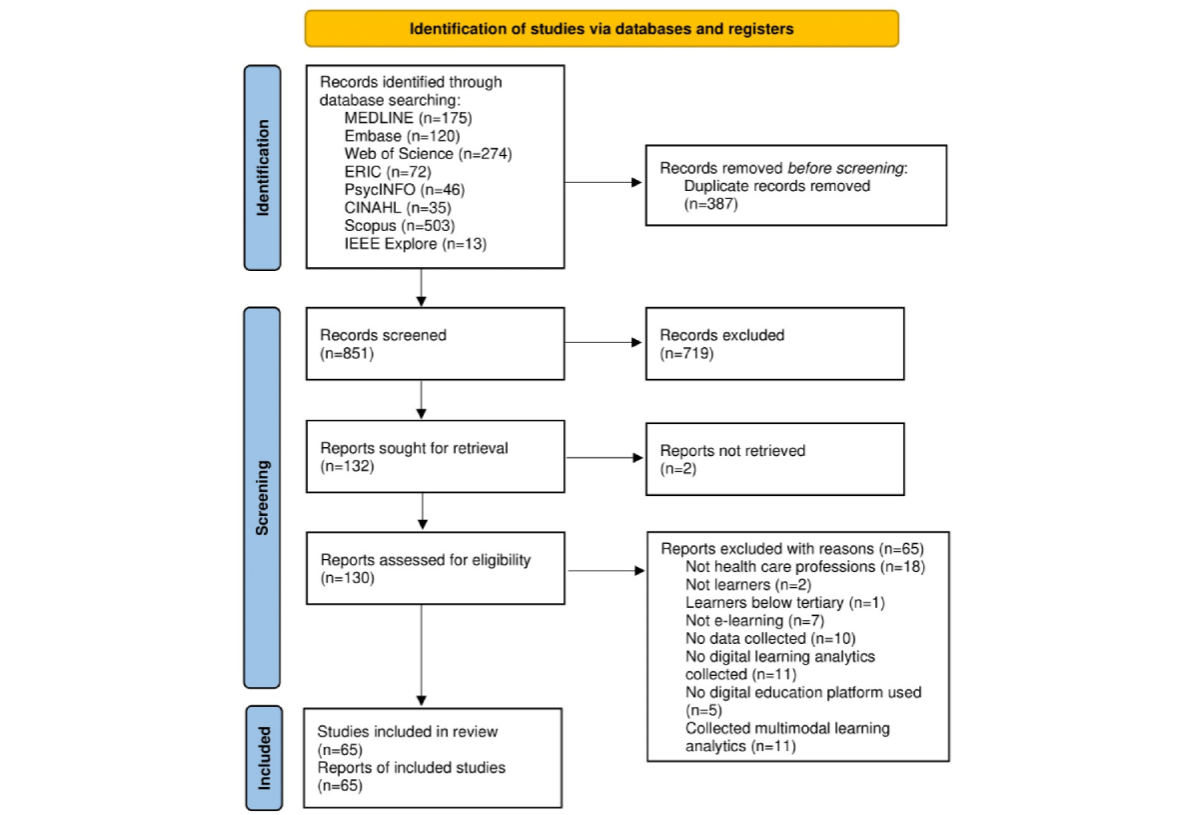
PRISMA-ScR (Preferred Reporting Items for Systematic Reviews and Meta-Analyses extension for Scoping Reviews) diagram of the literature search process.

The search yielded 1238 papers (Figure 1), of which 851 were obtained after deduplication; 719 were excluded after title and abstract screening because they did not meet the inclusion criteria, and another 2 were excluded because of the unavailability of full texts. Full texts of 130 papers were retrieved and screened, of which 65 met the inclusion criteria.

### Study Characteristics

Among the included papers, the number of articles showed an upward inclination over the last decade, reaching a relative maximum in 2020 (n=14) and declining in 2021 (n=7). The most common study locations were the United States (n=12), Australia (n=11), Saudi Arabia (n=10), and Spain (n=6). Other countries had ≤5 papers, and 2 papers did not report the study location. A total of 60 studies were conducted in a university context with undergraduate and postgraduate students, and the remaining studies recruited health care professionals. The most common subject areas were medicine (n=32), dentistry (n=8), and nursing (n=7). Moodle [23] was the most commonly used digital education platform among the included articles (n=29), followed by Blackboard (n=4) [24,25]. A summary of article characteristics is shown in Table S2 in Multimedia Appendix 1.

### Life Cycle of LA

**Figure 2:**
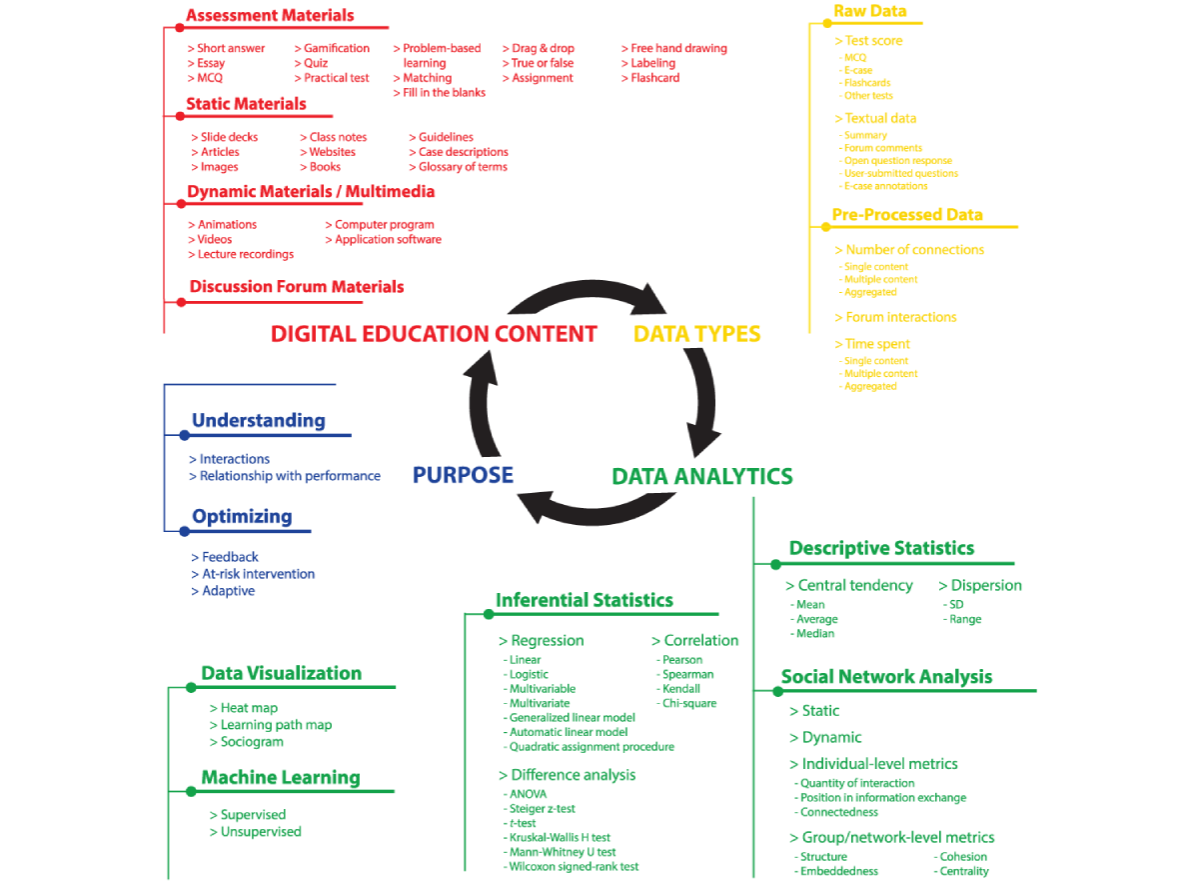
The conceptual map showing the whole life cycle of the learning analytics process. MCQ: multiple-choice question.

In our literature analysis, we identified several components of the LA process. We propose that these components can be conceptualized as part of a larger LA life cycle. Figure 2 shows the life cycle of LA, starting from digital education content creation, followed by the data collection process, the use of analytics to process the collected data, and finally the purposes for which LA was used. Digital education content is mostly created by educators to support student learning, but in certain cases, it can also be created by learners’ peers (eg, discussion forum materials). While students are interacting with the provided digital education content, data on their use are collected and stored for further processing. The *raw* data include the *click-level* data that contain time-stamped information about every interaction with the digital education platform. The preprocessed data refer to the data that are mostly aggregated and shown in a dashboard. Both types of data can be used later in the process of analyzing the data. We identified 5 types of analytical approaches: (1) descriptive statistics, (2) inferential statistics, (3) data visualization, (4) machine learning, and (5) social network analysis. Finally, as stated in the definition of LA itself [10], the purpose of LA is to “understand and optimize learning and the environments in which it occurs.” Once the entire process of collecting and analyzing data is complete, the knowledge gained can be used to improve the produced digital education content, which then starts a new round of the entire LA life cycle.

### Research Question 1: What Types of Content Are Used in LA Studies in Health Care Professions Education?

Education content is any material that can be used to facilitate the learning process. Currently, many types of content are available for various learning purposes. Exploring the various types of content used in health care professions education is essential for understanding the contextual complexity that shapes the way we can build and use LA. The contents of the various studies examined in this review can be classified into 4 broad categories: (1) static materials, (2) dynamic materials or multimedia, (3) discussion forum materials, and (4) assessment materials (Table S3 in Multimedia Appendix 1). A total of 62% (40/65) of studies reported 1 or 2 types of digital education content [3,24,26-63], 28% (18/65) of studies reported 3 types [25,64-80], and the remaining 11% (7/65) of studies reported all 4 types [81-87].

*Static materials* are any type of digital education content that does not contain interactive elements. These include articles [81], guidelines [25], class notes [64], websites [26], case descriptions [27], glossaries of terms [28], images [65], slide decks [66], and books [82]. A total of 45% (29/65) of papers discussed providing static materials and outlining what specific materials were available for learners to view or read [25-29,42,58,60,61,63-70,72-74,76-78,80-82,84-86]. Another 9% (6/65) of studies used static materials but did not specify what materials were prepared for learners [36,44,75,79,83,87]. Among the various options, class notes appeared to be the most popular static material. Static materials remained popular because they can be easily viewed on any browser or mobile device and do not require additional plug-ins or technology [29].

*Dynamic materials* or *multimedia* are types of content that combine visual (eg, images, text, and animations) and auditory materials (eg, audio and podcast) in a single content package to convey a message [30]. Typically, they consist of animations [68], videos [69], and lecture recordings [31]. These materials were found to be the second most popular format and were discussed in 49% (32/65) of papers [24-26,30-35, 46,47,52,56,57,64-71,74,76-78,80-82,84-86]. A total of 8% (5/65) of other studies did not specify which dynamic materials they had prepared for their learners [41,44,79,83,87]. Although videos remained the primary form of instruction in health care professions education, some studies have highlighted the importance of experiential learning [32]. A few studies have examined a computer program [33] or application software [71] with interactive controls and activities that provide learners with some level of control to create a sense of authenticity, immersion, and interaction. This would allow learners to contextualize theoretical knowledge in a simulated environment by linking new experiences to prior knowledge. This type of content is an excellent solution for training in health care professions in a safe environment without fear of distressing the patient [34]. Undeniably, dynamic materials or multimedia are more versatile and promise greater engagement in learning than static text or images alone [35].

*Discussion forum materials* are provided to conduct specific learning activities outside the classroom, which are structured by educators [36]. The discussion forum is one of the most important social elements in a digital education course [3]. It is an effective tool to transform the classroom into a networked learning environment [86]. Students can actively engage in their learning process and develop important communication skills while posting, commenting, liking, and collaborating with other students on course content [83]. In total, 35% (23/65) of papers presented the use of a discussion forum to support student learning, although the materials used in the papers were not specified [3,36-38,48-51,54,59,62,64,71-73,75,81-87]. Understandably, each forum has its own set of category-based threads that are visible to all the learners in a course. These threads or posts create an active learning environment in which students regularly reflect on course content and actively collaborate to build knowledge through discussion. As discussion forums are asynchronous, they provide a safe environment for students to share ideas and learn from each other [73].

*Assessment materials* were the most popular digital education content, with 72% (47/65) of papers discussing them [24,25,27-30,32-35,39-43,45,46,52,53,55,56,58,60,61,65-87]. Assessment materials are important for improving the overall quality of learning. Learners’ perceptions of when, how, and what they are assessed on affect how they learn. Assessment materials include drag and drop, fill in the blanks, true or false [81], matching, freehand drawing, labeling [68,74], flash cards [35,40], quizzes [75], multiple-choice questions (MCQs) [84], problem-based learning [85], practical tests [39], assignments [84], short answers [86], and essays [41]. Most assessment materials were administered at the end of a module or course so that educators could determine how much students have learned and retained and whether learning objectives have been met [4,25]. Of the 47 papers, 32 (68%) papers indicated that their assessment materials included feedback to help learners gain a better understanding of a topic and provide them with a reflection prompt [25,27,28,30,32-34,42,43,53,55,58,60,61 ,65-68,70-72,74,76,77,79-81,83-87]. Furthermore, assessment materials were also a fun and interactive way to create natural breaks between main topics and make learners feel like the course is progressing [65]. Although gamified assessments are possible, only 1 study has investigated this [43]. Students were found to be more motivated in the assessments when such features were effectively integrated.

### Research Question 2: What Types of Data Are Collected in Health Care Professions Education Through Content Engagement?

**Figure 3:**
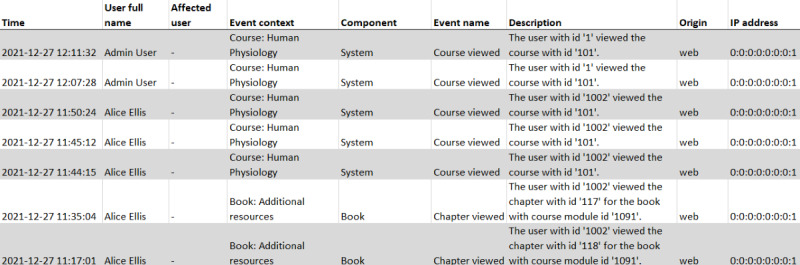
Example of a screenshot of a person’s log of actions performed on a Moodle course.

Data collected from the digital education platform can be broadly divided into 2 categories: raw and preprocessed data. Raw data consist of unprocessed data taken directly from the platform often in the form of log files. Many platforms automatically generate log files as a record of all user interactions with the system. For instance, the Moodle platform maintains a log of all user actions on the system and the time they were made (Figure 3). Preprocessed data can then be obtained by processing the raw data and aggregating data points to focus on a specific aspect of it. For example, the same log data can be used to determine how often a student viewed a given course by extracting each instance of a specific user accessing that course. We found that most preprocessed data types fit into the broader categories of (1) *number of connections*, (2) *time spent*, and (3) *forum interactions* (Table S4 in Multimedia Appendix 1). Moreover, not all raw data consist of log files. Other types of raw data can be collected directly from relevant learning materials on the digital education platform, which we categorized into (4) *test scores* and (5) *textual data*.

*A number of connections* refers to data that measures the number of times users connect to the digital education platform. This is one of the most common data types collected in LA research and can be derived from log data from many platforms. Of articles that collected data on the number of connections to the platform, 32% (21/65) of collected data on multiple content types individually [24,26,27,29,36,37,39,56,58,63-65,67, 73,76,78-80,82,85,86]. This allows for more detailed results by comparing the use of different digital education content types with student outcomes. Furthermore, 12% (8/65) of papers counted only the total, aggregated number of connections to the platform, without further dividing by content type [30,44,45,52,74,75,77,83], whereas 71% (25/65) of articles focused on connections to just 1 content type, such as in a study by Corrias and Cho [46], which measured student access to only the web-based video content [25,28,31,33,35,38, 40,42,43,46-51,54,55,57,59,61,62,68-70,72]. A total of 26% (17/65) of papers used log data to generate timeline analytics, deriving insights into the distribution of connections over a day, week, semester, or even school year [31,33,39-43, 47,53,55,57,65,68,70,71,75,86]. In so doing, researchers could determine the times at which materials were most frequently accessed and identify potential patterns, such as the relationship between material use and examination dates or the material access behaviors of higher-performing students.

*Time spent* measures the amount of time that users spend on the digital education platform. One way in which these data can be used is to understand how students interact with learning materials. For example, Cirigliano et al [65] measured the number of times students spent learning on each series of learning cards. They were able to distinguish between 3 types of student card interactions: those where students spent <20 seconds per card (rushing), those where they spent >100 seconds per card (idling), and those where they spent 20 to 100 seconds. Gaining a better understanding of rushing and idling can provide insights into whether students truly engage in digital education content. As with the number of connections, only 3% (2/65) of papers specified the amount of time spent on each content type [65,71], whereas 3% (2/65) of others calculated the overall time spent on the digital education platform [47,75]. The remaining (13/65, 20%) reviewed papers examined connections to only 1 content type [31,33,39-43,53,55,57,68,70,86].

*Forum interactions* consist of data derived from the students’ use of web-based forums [3,37,38,48-51,54,59,62,64]. The data collected typically included the IDs of the sender and receiver, the times that the post was written and modified, the post’s content, and the thread and forum where it was written. A total of 5% (3/65) of articles used forum interactions to observe how students engaged in problem-based learning that involved discussing concepts in web-based groups moderated by a tutor [48-50]. Forum interaction data were largely analyzed via social network analysis [51].

*Test scores* constitute data from assessments provided on a digital education platform. In most cases, researchers have collected the accuracy of participant performance in web-based clinical cases or e-cases [52,82]. The next largest group consisted of participants’ scores on the MCQs [41,43]. A total of 2% (1/65) of articles did not specify the type of assessment on which they collected data [75], and 2% (1/65) of articles featured not only MCQs but also fill in the blank, jumbled sentences, and matching questions [25]. In addition, 3% (2/65) of studies collected students’ performance on flash cards [35,40].

*Textual data*, such as written assessments, discussion forums, and email communications [88], can provide additional insights into the learning process, which are not captured by quantitative data. Among the papers reviewed, we identified 17% (11/65) of papers that digitally collected textual data [3,24,25,34,41,60,66,76,79,84,86]. The majority (6/11, 55%) were open question responses from web-based surveys [24,25,60,76,79,86]. Other types of textual data collected included forum comments [3,84], user-submitted questions [66], e-case summary statements [41], and e-case annotations [34]. Textual data were generally analyzed manually, such as in a study by Colthorpe et al [24], where the authors manually categorized open question response data based on an existing classification system. However, 5% (3/65) of articles used machine learning tools to analyze textual data [34,41,66]. For example, Harrak et al [66] used an automatic annotation tool to analyze questions submitted by students to a digital education platform. The tool identified keywords in student questions, weighed them, and divided the questions into different sentences.

### Research Question 3: What Approaches Are Used for Data Analytics in Health Care Professions Education?

We identified 5 analytic approaches used to process the collected data on students using digital education content (Table S5 in Multimedia Appendix 1): (1) *descriptive statistics*, (2) *inferential statistics*, (3) *data visualization*, (4) *machine learning*, and (5) *social network analysis*. Descriptive statistics summarize the characteristics of the collected data. Inferential statistics allow researchers to make predictions about the data [89]. Data visualization is used to present values visually [90]. Machine learning supports automated learning and makes predictions about collected data [91]. Social network analysis is the process of investigating social structures using the graph theory [76].

*Descriptive statistics* was the most commonly used approach to summarize the characteristics of the collected data: in 20% (13/65) of papers as the only approach [31,34,35,46,67,68,72-74,76,80,81,86] and in 89% (58/65) of papers as one of the used approaches [3,24-31,33-43, 45-56,58-63,65-68,70,72-74,76-87]. Descriptive statistics can be further broken down into measures of *central tendency* (eg, average, mean, and median) and *dispersion* (eg, range and SD). The average is defined as the sum of all numbers divided by the total number of values. The median is the middle number in a sorted list of numbers. Mean or average was more frequently used in the reviewed papers as a measure of central tendency than the median. The range is calculated as the maximum value minus the minimum value in the data set. SD measures the amount of data dispersion. Range and SD were almost equally used throughout the reviewed studies. Descriptive statistics measures were typically used to compare different student groups; for example, among the active, passive, and selective groups in a study by Ahmad et al [39]; English, Spanish, and French users in a study by Brands et al [81]; or different cohorts in a study by Alexander et al [67].

*Inferential statistics* used for hypothesis testing can be divided into *correlation*, *regression*, and *difference analysis*. This was the second most popular approach, used in 62% (40/65) of papers [24,25,29,30,33,36-41,43,45,47-56,58,59,62,63,65,66, 70,71,75,77-79,82-85,87]. This correlation indicates whether the 2 data types collected are related. Regression investigates the relationship between an outcome (ie, dependent variables) and independent variables. The purpose of the difference analysis is to test the differences between the means of variables. Inferential statistics can be divided into *parametric* and *nonparametric* tests. Parametric tests make assumptions about the parameters of the population distribution from which the sample is drawn, whereas nonparametric tests are distribution free.

A correlation coefficient (eg, Pearson, Kendall, and Spearman) is used to measure the strength of the relationship between numeric data types. For the Pearson correlation, both variables should be normally distributed. Kendall rank correlation and Spearman rank correlation do not carry any assumptions about the distribution of the data. Pearson correlation was used more often than Spearman correlation. Kendall rank correlation was only used in a study by Saqr et al [50]. Chi-square tests are used to determine whether nominal data types are associated. Chi-square tests were performed in a total of 15% (10/65) of papers [25,41,49,50,53,54,71,79,82,83]. The correlation was typically used to determine the variation in different data types collected through digital education platforms (eg, number of log-ins, total time, or time per question) and examination performance (eg, final grade) [29,55] or course completion [83].

Regression analysis can be divided into *linear* and *logistic* regression, depending on whether the outcome is continuous or dichotomous, or *multivariable* and *multivariate*, depending on whether there is 1 outcome or ≥1 outcome. Linear regression was used slightly more often than logistic regression. In addition to multivariate and multivariable regressions, generalized linear model was used in studies by Cirigliano et al [65] and Mu et al [53], automatic linear model was used in studies by Perumal et al [75] and Saqr et al [50], whereas the quadratic assignment procedure logistic regression was used in a study by Saqr and Montero [38]. Throughout the reviewed papers, the authors used collected data, such as age and attendance [25] and videos and quizzes accessed [56], as independent variables to predict dependent variables, such as student accuracy on the MCQ as a general indicator of successful engagement [65] or for the prediction of at-risk students [75].

Difference analysis or mean differences can be parametric (eg, *t* test or ANOVA) or nonparametric (eg, Mann-Whitney *U* test or Wilcoxon signed-rank test). In the papers, parametric tests were used in 22% (14/65) of instances [24,30,43,47,53-55,58,71,77,78,84,85,87], whereas nonparametric tests were used 14% (9/65) of the times [24,39,45,52,55,66,77,78,83]. Among parametric tests, ANOVA and *t* tests were used almost equally frequently. Mann-Whitney *U* test was more frequently used as a nonparametric test than others. For example, difference analysis was used to show substantial differences between an experimental group using a Smart Tutoring System in Moodle and a control group that did not use it [77] or showed that the students’ use of digital education was associated with their subjects’ final status (ie, success or failure) [78].

Data visualization techniques used in the analyzed papers can be divided into heat maps, learning path maps, and sociograms. Heat maps, used in 11% (7/65) of different studies, are visual representations of data that use color depth to make it more intuitive to interpret and evaluate the data [24,33,52,53,57,60,79]. Learning path maps, used to present the collected data in 11% (7/65) of papers, helped to visualize different course units that students master during a specific subject or program [28,29,32,33,37,42,69]. A sociogram is a visual representation of a person’s social connections. It was used in addition to other social network analysis techniques in 11% (7/65) of studies [38,48-51,54,59].

*Machine learning* approaches consist of 2 types: *supervised* and *unsupervised*. Supervised learning makes use of labeled data sets to train or *supervise* algorithms so that they can accurately identify data or forecast outcomes. In the reviewed papers, supervised machine learning was mostly used for classification tasks; for example, decision tree analysis was used to understand the relationship between the initially selected diagnostic hypotheses while examining web-based patient’s cases and the final submitted hypothesis [27], support vector machines was used to predict students’ final results in blended learning courses using student access time series generated from Moodle log [44], and naive Bayes network was used to provide students with feedback on the quality of their asked questions [66]. Unsupervised learning analyzes and clusters unlabeled data sets that can be used to identify hidden patterns in the data. k-means was the most popular unsupervised algorithm used in 9% (6/65) of papers, mostly as a mechanism for the early detection of at-risk students [24,26,58,66,77,79].

*Social network analysis* can be divided into individual-level and group/network-level measures and can be either static or dynamic. Individual-level measures are calculated on the level of an individual (ie, a student). Group or network-level data represent a group of students or the whole class (ie, the network). Individual-level measures can be further divided into a quantity of interaction, position in information exchange, and connectedness. Group or network-level measures included centrality, structure, embeddedness, and cohesion.

The quantity of interaction denotes how many interactions a student had in total (ie, degree centrality). One can also separately examine a measure of popularity (ie, in-degree centrality) or a measure of outgoing interaction diversity (ie, out-degree centrality). Position in information exchange (eg, betweenness centrality, information centrality, and closeness centrality) defines the role of students in information exchange in comparison with other students. Connectedness (eg, eigenvector centrality and clustering coefficient) denotes the proportion of the theoretical number of connections that have been achieved. Individual-level measures were calculated in almost all papers, except in a study by Jan and Vlachopoulos [3], where the authors focused only on calculating group-level measures, which were used for community detection.

Centrality measures denote the distribution of centrality measures within the group or network. The structure of the graph includes node count (ie, the number of students in each network), edge count (ie, the number of interactions), the average distance between all pairs of nodes, and average degree (ie, the average number of edges per node). Embeddedness includes network density and reciprocity. Network density is the proportion of actual interactions to the greatest feasible ratio among all members. Reciprocity is the ratio of reciprocated edges when 2 individuals exchange their responses. Cohesion includes efficiency, vulnerability, and transitivity. Efficiency is a valuable indicator of a network’s robustness and tolerance to limited failures or the removal of a small number of members. Vulnerability is a measure of graph sensitivity to information flow disturbance. Transitivity is the tendency of 2 individuals who have a common connection and a structural network characteristic that appears as triangles of nodes. Group-level measures were calculated in all 14% (9/65) of papers that used social network analysis [3,38,48-51,54,59,62].

In the reviewed papers, forum interactions were used as a type of data for building social networks to examine the properties of community-based [3] and problem-based learning [49,51]. More specifically, students’ positions, interactions, and relationships in a network were used to predict their final performance. The most important predictors were information centrality and eigenvector centrality [50]. It was also shown that larger groups are associated with the decreased performance of individual students, as a high group size led to a less cohesive group, with less information exchange among students [48]. On the other hand, the well-performing groups were characterized by active and reciprocal interactions among students and group cohesion measures (transitivity and reciprocity) [59].

### Research Question 4: What Are the Purposes of Analyzing the Collected Data in Health Care Professions Education?

The purposes for which LA data were used in the literature were (1) *understanding* and (2) *optimizing* learning and the contexts in which it occurs (Table S6 in Multimedia Appendix 1). Under the category of understanding, the purpose of LA can be further divided into the subcategories of understanding user *interactions* with the digital education platform and understanding the relationship between those interactions and user *performance*. The category of optimizing can also be subdivided into the subcategories of providing *feedback*, *at-risk intervention*, and *adaptive learning.*

*Understanding interactions* involves conducting LA to understand how students use the digital education platform. This could be as simple as examining the mean number of connections to a given material or as complicated as modeling how students make decisions in an e-case task. The interactions subcategory comprised the largest group in the papers we reviewed, with 91% (59/65) of papers examining patterns in student use of the platform [3,24-47,49,50, 52,53,55-58,60-62,64-74,76-87]. Although more than a half of the papers (36/59, 61%) had at least one other purpose of understanding aside from understanding the interactions [24,25,29,30,33,34,36-41,43,44,47,49,50,52,53,55,58,62,64-68,71,77-79,82-86], 39% (23/59) of the papers focused only on understanding the interactions [3,26-28,31,32,35,42,45,46,56,57,60,61,69, 70,72-74,76,80,81,87]. For instance, Brands et al [81] used a combination of user statistics from the course digital education platform and student course evaluations to gauge student use of and satisfaction with a web-based course. On a larger scale, Kim and Kim [80] aggregated LA data from 36 medical schools in Korea to examine trends in student use of digital education content.

*Understanding performance* involves the investigation of the relationship between learners’ interactions with the digital education platform and their performance. Performance can be evaluated in terms of student knowledge, skills, and attitudes. By examining the relationship between LA data and performance variables, authors can make conclusions about whether the use of digital education content leads to better outcomes and, if so, which type of content is more beneficial to students than others. As the second-largest category, a total of 65% (42/65) of papers fell under this group [24,25,29,30,33,34,36-41,43,44,47-55,58,59,62-68,71,75,77-79,82-86]. For example, Ahmad et al [39] used data on students’ patterns of digital education content use to group students according to their time management strategies. They then examined the relationship between these time management strategies and students’ academic performance.

*Feedback*, under the category of *optimizing* learning, involves the use of LA data to directly provide information to students about their learning. All articles (6/65, 9%) identified in this category provided feedback based on student performance on e-tests [33,34,53,55,58,77]. The feedback itself consisted simply of whether the students’ responses were correct, often accompanied by a further explanation of the correct answer. For example, Sáiz-Manzanares et al [58] provided learners with feedback on web-based patient cases with MCQs about theoretical knowledge as well as actions that should be taken to treat the patient. Once the learner selected an answer, they received feedback indicating whether it was correct as well as the appropriate actions that needed to be taken for the patient (eg, “You should call a cardiology consultant immediately”) and an explanation of the correct answer. In addition, hints were provided to guide students in performing the correct action.

*At-risk intervention* aims to provide additional support to students determined to be *at risk* of, for example, failing a course. To determine a student’s risk, educators must first construct a predictive model that describes the relationship between predictors (eg, scores on formative assessments) and course failure. We identified 20% (13/65) of papers where the authors stopped short of presenting a model that could be used to predict student performance in the future [34,37,38,44,50,54,55,58,59,66,72,75,79]. However, only 1 study has put this predictive model into practice by providing interventions, namely, in a study by Sáiz-Manzanares et al [79], the authors constructed a model of students’ risk of dropping out of the course based on which course educators provided personalized tutoring to at-risk students.

*Adaptive learning* in the educational context involves a system in which learners’ interactions with digital content determine, at least partly, the nature of future content delivered to them [92]. In this review, we found only 6% (4/65) of instances of adaptive learning [29,30,40,57]. As with papers in the feedback subcategory, most of these studies used test scores as the basis for determining subsequent digital content. In the study by Linden et al [30], when students answered questions incorrectly, they were provided with a set of explanatory content, after which they had the opportunity to answer the same question again before being shown the next topic. In a study by Liu et al [29], the authors implemented a more involved form of adaptive learning: learners took a diagnostic test at the start of the module based on which the digital education platform created a personalized learning path and study plan for the student. Moreover, students could use the Test Me function to test themselves again, which would cause corresponding changes in their study plan. Finally, the platform mentioned in a study by Menon et al [40] used a combination of learner accuracy and confidence on test questions to space the repetition of test items. An exception to this trend could be seen in a study by Gilliland [57], where the authors used the frequency of student engagement with particular topics on the learning platform as a proxy for the perceived difficulty of said content. They then communicated these findings to educators so that more teaching time could be allocated to challenging topics.

## Discussion

### Principal Findings

#### Content Used in LA Studies

Digital education has grown to be a critical component of higher education [3]. In the training of health care professionals, this review discovered that a variety of content types are used to achieve different types of outcomes (ie, knowledge, skills, and attitudes) [93]. The content can be text or audio, static or dynamic and in the form of video or animation [32,58,73,78]. Adding to the complication, the same content can be used by several users at different times and under different conditions. These factors make the standardization of data collected from various contexts extremely difficult. This also leads to associated difficulties in creating LA from preprocessing to analysis. Similar findings have been documented in some previous studies, indicating how difficult it is to generalize predictive models across different courses because of the differences in course designs [18]. These findings suggest that relevant metadata about the pedagogical purpose of various types of content should be ideally captured in the future to support the development of LA that can be built, deployed, and interpreted across different contexts [94].

Another issue is the creation of valid and reliable measures for various types of outcomes based on trace data used across different types of content [94,95]. Given the complexities of learning and teaching contexts, it is important to understand how different types of content can be used to support different pedagogical purposes [30,40]. Although current LA tools provide educators with some level of insight into how students interact with content, these tools do not provide educators with enough actionable insights to inform decisions about how to design personalized learning experiences for their students. As such, future research should focus on ways to improve our understanding of how learners interact with various types of content as well as develop better measures for various types of outcomes based on traced data.

In addition, it is worth noting that even when metadata about pedagogical intent are collected or when all the courses follow the same nominal pedagogy (eg, problem-based learning), developing a generalizable predictive model might not be so easy, as we often miss collecting data about individual differences among students. This has been shown in a recent paper with data from several years of problem-based learning courses of a cohort of students enrolled in a medical school [96]. For example, some students are more likely to collaborate in group projects, whereas others prefer individual tasks; some students may spend more time reviewing their works before submission, whereas others submit their work immediately after completion; some students may tend to ask questions, whereas others do not; and some students are quick learners, whereas others require more efforts and time [14,46,80,82]. Therefore, it is critical to consider the individual differences among students when developing predictive or generalized models for LA.

#### Data Collection

LA, in its purest form, *passively* collects data from only *1* source. In contrast to LA, in multimodal LA, data are collected and integrated from different sources [97]. The purpose of multimodal LA is to *collect, synchronize, and analyze data from different communication modalities to provide on-time feedback* [98]. Using multimodal LA in health care professions education would allow for a more panoramic understanding of the learning processes and the different dimensions related to learning (ie, knowledge, skills, and attitudes). Moreover, although LA collects data about students seamlessly from digital education platforms, which makes it objective, it fails to capture individual differences and internal conditions of the learners (eg, their motivation, self-efficacy, cognitive load, or affective states) [99,100]. There is abundant evidence in the literature that showed the importance of motivational constructs as facilitators of academic self-regulation and achievement [101,102].

In the reviewed literature, there were a few studies in which the authors used multimodal LA to collect data while evaluating students’ knowledge, skills, and attitudes. Health care professionals’ *attitudes* toward learning were collected during their team-based learning session with videos and microphones in a study by Chua et al [103]; wearable sensors for heart rate, electrodermal activity, electroencephalography signals in studies by Antoniou et al [104,105]; and the Raspberry Pi minicomputer and Polar H10 chest belt in a study by Li et al [106]. Student *skills* were assessed by simulators in studies by Kennedy et al [107] and Chiu et al [108] while performing surgical tasks and by Microsoft Kinect in a study by Di Mitri et al [109] while performing cardiopulmonary resuscitation training. Finally, the gaze-following framework presented in a study by Barmaki and Guo [110] was used to track how *knowledge* of anatomy was gained in a collaborative effort. The aforementioned studies showed how multimodal LA can help collect data on all 3 components of learning separately. However, it is of vital importance to develop a comprehensive framework in which the learning of health care professions can be simultaneously tracked through all 3 prisms of learning, both in the digital and physical worlds.

A large amount of prior research has focused on self-report instruments used to measure self-regulation [111-113]. However, self-report instruments (eg, questionnaires, interviews, or learning diaries [114]) rely heavily on memories of the experience, where memories are biased and incomplete [115]. In addition, these instruments do not describe how learners dynamically adapt and modify their behavior during the actual learning process [116,117]. Previous research has shown that trace-based data collected from digital education platforms can reflect self-reported data to a certain degree [118]. Although research on trace-based self-report measures is promising [99,119], the collection should be accompanied by incentives for students to self-report at the time when they complete their activities to avoid problems with conventional measures, which are usually not collected in real time [12]. An example of how this can be done is by collecting students’ clicks on a 2D canvas [120]. In this sense, the 2D canvas would be used to report on self-efficacy and cognitive load for each activity in the course, while that click would also become a bookmark allowing students to easily retrieve activities that they felt unsure about or found difficult.

#### Data Analytics

LA data are collected in real time, but data analytics is usually performed after course completion. The advantage of conducting real-time analytics is, for example, to implement early detection of at-risk students. Unfortunately, only a handful of studies have investigated how the detection of students at risk in combination with a personalized educational response to each student can be used to minimize students’ dropout rates [58,79]. The second detected gap is that LA data are mostly collected over a period of up to 1 year or for 1 cohort of students (eg, 10 months in a study by Cirigliano et al [65], 1 academic year in a study by Koh et al [87], or a cohort of undergraduate students in a study by Kuhbeck et al [55]). Even when the data are collected for a longer period, they are mostly either jointly analyzed (eg, 3 years of data in a study by Brands et al [81]) or the comparison is made among different years (eg, 2 consecutive cohorts in a study by Chan et al [56]) or different courses (eg, 4 different courses in a study by Saqr et al [49]) without any changes made between 2 consecutive years or cohorts.

In cases where changes are implemented between 2 consecutive years or cohorts, the decision of what to change is very often not based on data analytics performed on the LA data previously collected. For example, in a study by Alvarez-Mendez et al [82], the authors introduced new tools, such as a glossary, a quiz, and a wiki, without relying on data analytics results. When comparing the performance of students before and after the aforementioned changes, Alvarez-Mendez et al [82] did not find significant relationships between the final grades and the digital education content provided. Among the reviewed papers, we identified only 1 paper [86] in which student feedback collected during the previous run was used as a tool for improving the quality of course content in subsequent years or cohorts. Thus, we propose that student feedback on digital education content or how teaching is conducted is used to improve the learning experience for subsequent years or cohorts. This would result in the closing of the LA life cycle loop.

Not only is LA offering valuable insights to educators on their students and courses, but it is also providing useful information to higher education institutions. This information could be potentially used to inform strategic decision-making regarding achieving educational excellence. However, we acknowledge that institutional planning and strategic decision-making processes are not easy tasks. This might be a consequence of a lack of understanding of (1) institutional culture within higher education, (2) the degree to which individuals and cultures resist innovation and change, or (3) approaches to motivating social and cultural change [121,122]. Indeed, in many studies, it has been found that the most significant challenges that confront LA adoption are not technical but of a social nature [123-125]. In this context, and in the absence of a strategic goal or vision, LA data reporting has little power to start any significant changes.

#### Purposes

Among the purposes for which LA can be used, most of the studies we reviewed used LA to understand learning, either to simply understand learners’ interactions with the digital education platform or to determine the relationship between those interactions and learners’ eventual performance. However, the measurement of performance tended to be limited to end-of-course examination results. Although these insights may be useful for course educators and administrators, they provided a limited picture of how these students would eventually perform as health care workers. One way to address this gap is to use other measures of performance, such as textual data and the aforementioned multimodal LA [126]. In addition, a longitudinal study that follows learners into clinical practice would provide uniquely pertinent insights into the effectiveness of digital education content in health care worker performance. This would allow the assessment of not only learners’ theoretical knowledge but also their practical skills, as well as qualities such as communication with patients.

The second component, which was underrepresented in the reviewed studies, was the use of LA-derived insights to provide feedback, interventions, or adaptive learning. Of the 3 types of learning, feedback and interventions can both be considered as providing supplementary content to learners on a need basis, whereas adaptive learning can affect core course content as well by determining what modules a student is shown, and in which order, to suit the needs of the specific learner. However, we found few examples of researchers using LA-derived insights to fundamentally alter core course content or instructional design. One study of relevance was performed by Blakemore et al [84], who made iterative changes to a course’s design over several runs to evaluate the efficacy of learning activities on student performance.

A similar dearth of research using LA to intervene in the learning environment has been noted in the general literature on LA [127]. The potential of harnessing LA to assess the effects of alterations in course content on student performance and engagement is a promising avenue explored in nonmedical areas [128]. Previous work has demonstrated the effectiveness of LA-based personalized feedback tools for improving such outcomes as learning effectiveness [129], student motivation [130], and academic performance [131]. The use of personalized feedback provision systems, such as *OnTask*, could present a promising and cost-effective avenue for health professions education to pursue in the future [132].

### Strengths and Limitations

Although this scoping review was conducted according to the scoping review methodology, some limitations are worth noting. This review was guided by a formal protocol. To ensure a broad search of the literature, the search strategy included 10 databases; however, our inclusion criteria limited our review results to only English-language articles and published peer-reviewed literature from January 2010 to October 2021. Furthermore, as the data were extracted by 6 reviewers, we used the standardized extraction form to ensure consistency among reviewers. Nevertheless, the extraction process might be slightly affected by the presence of multiple reviewers. A final limitation is the rapid growth of the field; therefore, it is important to acknowledge that this scoping review is a snapshot at a particular point in time. Unlike traditional systematic reviews guided by well-defined constructs, in scooping reviews, it might be unfeasible to screen and synthesize all relevant literature on an emergent topic [133]. As our purpose was to primarily understand what LA techniques have been used in the education of health care professionals, our efforts to identify all eligible studies were limited in some respects.

### Practical Implications and Future Work

Our findings highlighted the need to develop an LA-specific implementation framework, drawing on empirical research related to LA efforts and drawing on existing knowledge and experience within the implementation science community. LA should be at the center of designing and redesigning courses for health care professionals. Namely, planning how LA should support the evolution of a course should come before the course starts rather than at its end. Finally, there is no need to repeat the mistakes that have already been made, and there is an opportunity to use the practical insights from existing research to provide an evidence-based foundation that can accelerate the implementation of LA for the development of professional health courses.

### Conclusions

In this paper, we reviewed 65 papers in which the authors used LA to collect data on health care professions in their classes. From these papers, we extracted some general characteristics of the whole LA process and proposed a framework for the whole LA life cycle that is field independent, meaning it can be used beyond health care professions education. We then used the proposed framework to review the 65 papers.

Our study is student-centric, although we acknowledge that, in addition to students, there are other stakeholders such as educators and institutions involved in the entire LA process. We focused on students because most of the reviewed papers focused on students. This is consistent with the findings of Lee and Recker [133], where out of 47 reviewed papers, only 3 studies did not focus, at least partly, on students.

Finally, we identified the gaps in the current literature and proposed how they could be closed. The main limitation of almost all the papers we reviewed is that they did not use LA to iteratively inform course design and redesign. The purpose of collecting and analyzing LA data was mostly for an exploratory analysis or predictive analysis conducted within the borders of the same year or cohort. This means that the results of data analytics were not used to improve subsequent classes but rather to make some predictions about students who had already finished that class. In this sense, the potential of LA in health care professions education is not fully used.

## Appendix

Multimedia Appendix 1 Tables listing extracted characteristics for each research question.

Multimedia Appendix 2 Search strategies for all included databases.

## Abbreviations

LA: learning analytics
MCQ: multiple-choice question
PRISMA-ScR: Preferred Reporting Items for Systematic Reviews and Meta-Analyses extension for Scoping Reviews

## Data Availability

All relevant data are included in this publication. The titles of the papers included in this review are identified in the tables and Multimedia Appendices 1 and 2. All excluded papers are available on request from the corresponding author. Researchers who require further details may contact the corresponding author with a methodologically sound proposal.
