# Supplementary material for: Empowering Health Care Education Through Learning Analytics: In-depth Scoping Review
Source: J Med Internet Res. 2023 May 17;25:e41671. doi: 10.2196/41671 (PMC10233437; doi:10.2196/41671)
Supplement: Multimedia Appendix 2 [file jmir_v25i1e41671_app2.docx]

**MEDLINE (OVID interface) Search Strategy**

1. exp biomedical engineering/ or exp chiropractic/ or exp dentistry/ or exp dietetics/ or exp optometry/ or exp orthoptics/ or exp pharmacology/ or exp pharmacy/ or exp podiatry/ or exp psychology, medical/ or exp radiography/ or exp serology/ or exp anatomists/ or exp coroners and medical examiners/ or exp dental staff/ or exp dentists/ or exp specialization/ or exp surgical procedures, operative/ or exp infection control practitioners/ or exp pharmacists/ or exp physicians/ or exp education, predental/ or exp education, premedical/ or exp education, professional/ not education, veterinary/ or exp evidence-based practice/ or exp health educators/ or exp medicine/ or exp nursing/ or exp allied health occupations/ or exp health occupations/ or exp allied health personnel/ or exp health personnel/ or exp medical laboratory personnel/ or exp medical staff/ or exp nurses/ or exp nursing staff/ or exp personnel, hospital/ or exp students, health occupations/
2. (clinic* or dent* or dietetic* or dieti?ian* or doctor* or general practioner* or health* or hospital administrator* or laborator* or medic* or nurs* or occupational* or pharmacist* or physical therap* or physician* or physiotherap* or predent* or premedic* or therap*).tw,kf
3. (an?esthesi* or cardiolog* or dermatolog* or gyn?ecolog* or neurolog* or nutrition* or obstetric* or oncolog* or orthodont* or orthopti* or p?ediatric* or patholog* or podiatr* or psychiatr* or psycholog* or radiolog* or rheumatolog* or surg* or urolog* or midwi?e*).tw,kf. adj3 (clerk* or educat* or fellow* or graduate* or internship* or novice* or personnel or practitioner* or residen* or staff or student* or train* or tutor* or undergraduate*).tw,kf.
4. or/1-3
5. exp education, distance/ or exp educational technology/ or exp models, anatomic/ or exp patient simulation/ or exp Computer-Assisted Instruction/ or exp Data Collection/ or exp Data Mining/ or exp Educational Measurement/ or exp Multimethod Studies/ or exp Problem-Based Learning/ or exp Self-Directed Learning as Topic/ or exp Simulation Training/
6. ((adaptive or blended or collaborative or computer-based or computer-mediated or distance or online or personalized or practical or problem-based or project-based or self-directed or self-regulated or team-based or technology-enhanced or ubiquitous or visual) adj2 (learning or education)).tw,kf.
7. (elearn* or e-learn* or flipped classroom* or mlearn* or m-learn* or patient simulat* or virtual patient* or technology enhanced learning or massive open online course?).tw,kf.
8. (Aquifer or BioWord or Blackboard or canvas network or coursera or coursesites or edx or e-MedEdu or Equella or futurelearn or Hypocampus or iversity or Kaizen or McPeer or MedU or miriada x or Moodle or novoed or open2study or openlearning or Osmosis or pingpong or plato or Smart Sparrow or spoc or udacity or Vquest).tw,kf.
9. ((technologically enhanced or computer-* or medical school or adaptive or digital or virtual or online or e- or blended) adj2 (learning or education*) or online assessment or intelligent tutoring or smart tutoring or e-learning management or learning management or learning content management) adj2 (dashboard* or environment* or platform* or portal* or software or system*).tw,kf.
10. or/5-9
11. (learning analytics or academic analytics or educational analytics or educational data mining).tw,kf.
12. and/4,10-11
13. limit 12 to (english language and yr="2010 - 2021")

**Embase (OVID interface) Search Strategy**

1. exp biomedical engineering/ or exp chiropractic/ or exp dentistry/ or exp dietetics/ or exp optometry/ or exp orthoptics/ or exp pharmacology/ or exp pharmacy/ or exp podiatry/ or exp psychology, medical/ or exp radiography/ or exp serology/ or exp anatomists/ or exp coroners and medical examiners/ or exp dental staff/ or exp dentists/ or exp specialization/ or exp surgical procedures, operative/ or exp infection control practitioners/ or exp pharmacists/ or exp physicians/ or exp education, predental/ or exp education, premedical/ or exp education, professional/ not education, veterinary/ or exp evidence-based practice/ or exp health educators/ or exp medicine/ or exp nursing/ or exp allied health occupations/ or exp health occupations/ or exp allied health personnel/ or exp health personnel/ or exp medical laboratory personnel/ or exp medical staff/ or exp nurses/ or exp nursing staff/ or exp personnel, hospital/ or exp students, health occupations/
2. (clinic* or dent* or dietetic* or dieti?ian* or doctor* or general practioner* or health* or hospital administrator* or laborator* or medic* or nurs* or occupational* or pharmacist* or physical therap* or physician* or physiotherap* or predent* or premedic* or therap*).tw,kf
3. (an?esthesi* or cardiolog* or dermatolog* or gyn?ecolog* or neurolog* or nutrition* or obstetric* or oncolog* or orthodont* or orthopti* or p?ediatric* or patholog* or podiatr* or psychiatr* or psycholog* or radiolog* or rheumatolog* or surg* or urolog* or midwi?e*).tw,kf. adj3 (clerk* or educat* or fellow* or graduate* or internship* or novice* or personnel or practitioner* or residen* or staff or student* or train* or tutor* or undergraduate*).tw,kf.
4. or/1-3
5. exp education, distance/ or exp educational technology/ or exp models, anatomic/ or exp patient simulation/ or exp Computer-Assisted Instruction/ or exp Data Collection/ or exp Data Mining/ or exp Educational Measurement/ or exp Multimethod Studies/ or exp Problem-Based Learning/ or exp Self-Directed Learning as Topic/ or exp Simulation Training/
6. ((adaptive or blended or collaborative or computer-based or computer-mediated or distance or online or personalized or practical or problem-based or project-based or self-directed or self-regulated or team-based or technology-enhanced or ubiquitous or visual) adj2 (learning or education)).tw,kf.
7. (elearn* or e-learn* or flipped classroom* or mlearn* or m-learn* or patient simulat* or virtual patient* or technology enhanced learning or massive open online course?).tw,kf.
8. (Aquifer or BioWord or Blackboard or canvas network or coursera or coursesites or edx or e-MedEdu or Equella or futurelearn or Hypocampus or iversity or Kaizen or McPeer or MedU or miriada x or Moodle or novoed or open2study or openlearning or Osmosis or pingpong or plato or Smart Sparrow or spoc or udacity or Vquest).tw,kf.
9. ((technologically enhanced or computer-* or medical school or adaptive or digital or virtual or online or e- or blended) adj2 (learning or education*) or online assessment or intelligent tutoring or smart tutoring or e-learning management or learning management or learning content management) adj2 (dashboard* or environment* or platform* or portal* or software or system*).tw,kf.
10. or/5-9
11. (learning analytics or academic analytics or educational analytics or educational data mining).tw,kf.
12. and/4,10-11
13. limit 12 to (english language and yr="2010 - 2021")

# CINAHL, PsycINFO & ERIC (Ebscohost Interface)

S1 (MH "biomedical engineering"+) OR (MH chiropractic+) OR (MH dentistry+) OR (MH dietetics+) OR (MH optometry+) OR (MH orthoptics+) OR (MH pharmacology+) OR (MH pharmacy+) OR (MH podiatry+) OR (MH "psychology, medical"+) OR (MH radiography+) OR (MH serology+) OR (MH anatomists+) OR (MH "coroners and medical examiners"+) OR (MH "dental staff"+) OR (MH dentists+) OR (MH specialization+) OR (MH "surgical procedures, operative"+) OR (MH "infection control practitioners"+) OR (MH pharmacists+) OR (MH physicians+) OR (MH "education, predental"+) OR (MH "education, premedical"+) OR (MH "education, professional"+) NOT (MH "education, veterinary") OR (MH "evidence-based practice"+) OR (MH "health educators"+) OR (MH medicine+) OR (MH nursing+) OR (MH "allied health occupations"+) OR (MH "health occupations"+) OR (MH "allied health personnel"+) OR (MH "health personnel"+) OR (MH "medical laboratory personnel"+) OR (MH "medical staff"+) OR (MH nurses+) OR (MH "nursing staff"+) OR (MH "personnel, hospital"+) OR (MH "students, health occupations"+)

S2 ((TI clinic* OR AB clinic* OR SU clinic*) OR (TI dent* OR AB dent* OR SU dent*) OR (TI dietetic* OR AB dietetic* OR SU dietetic*) OR (TI dieti#ian* OR AB dieti#ian* OR SU dieti#ian*) OR (TI doctor* OR AB doctor* OR SU doctor*) OR (TI "general practioner*" OR AB "general practioner*" OR SU "general practioner*") OR (TI health* OR AB health* OR SU health*) OR (TI "hospital administrator*" OR AB "hospital administrator*" OR SU "hospital administrator*") OR (TI laborator* OR AB laborator* OR SU laborator*) OR (TI medic* OR AB medic* OR SU medic*) OR (TI nurs* OR AB nurs* OR SU nurs*) OR (TI occupational* OR AB occupational* OR SU occupational*) OR (TI pharmacist* OR AB pharmacist* OR SU pharmacist*) OR (TI "physical therap*" OR AB "physical therap*" OR SU "physical therap*") OR (TI physician* OR AB physician* OR SU physician*) OR (TI physiotherap* OR AB physiotherap* OR SU physiotherap*) OR (TI predent* OR AB predent* OR SU predent*) OR (TI premedic* OR AB premedic* OR SU premedic*) OR (TI therap* OR AB therap* OR SU therap*))

S3 ((TI an#esthesi* OR AB an#esthesi* OR SU an#esthesi*) OR (TI cardiolog* OR AB cardiolog* OR SU cardiolog*) OR (TI dermatolog* OR AB dermatolog* OR SU dermatolog*) OR (TI gyn#ecolog* OR AB gyn#ecolog* OR SU gyn#ecolog*) OR (TI neurolog* OR AB neurolog* OR SU neurolog*) OR (TI nutrition* OR AB nutrition* OR SU nutrition*) OR (TI obstetric* OR AB obstetric* OR SU obstetric*) OR (TI oncolog* OR AB oncolog* OR SU oncolog*) OR (TI orthodont* OR AB orthodont* OR SU orthodont*) OR (TI orthopti* OR AB orthopti* OR SU orthopti*) OR (TI p#ediatric* OR AB p#ediatric* OR SU p#ediatric*) OR (TI patholog* OR AB patholog* OR SU patholog*) OR (TI podiatr* OR AB podiatr* OR SU podiatr*) OR (TI psychiatr* OR AB psychiatr* OR SU psychiatr*) OR (TI psycholog* OR AB psycholog* OR SU psycholog*) OR (TI radiolog* OR AB radiolog* OR SU radiolog*) OR (TI rheumatolog* OR AB rheumatolog* OR SU rheumatolog*) OR (TI surg* OR AB surg* OR SU surg*) OR (TI urolog* OR AB urolog* OR SU urolog*) OR (TI midwi#e* OR AB midwi#e* OR SU midwi#e*)) N3 ((TI clerk* OR AB clerk* OR SU clerk*) OR (TI educat* OR AB educat* OR SU educat*) OR (TI fellow* OR AB fellow* OR SU fellow*) OR (TI graduate* OR AB graduate* OR SU graduate*) OR (TI internship* OR AB internship* OR SU internship*) OR (TI novice* OR AB novice* OR SU novice*) OR (TI personnel OR AB personnel OR SU personnel) OR (TI practitioner* OR AB practitioner* OR SU practitioner*) OR (TI residen* OR AB residen* OR SU residen*) OR (TI staff OR AB staff OR SU staff) OR (TI student* OR AB student* OR SU student*) OR (TI train* OR AB train* OR SU train*) OR (TI tutor* OR AB tutor* OR SU tutor*) OR (TI undergraduate* OR AB undergraduate* OR SU undergraduate*))

S4 (MH "education, distance"+) OR (MH "educational technology"+) OR (MH "models, anatomic"+) OR (MH "patient simulation"+) OR (MH "Computer-Assisted Instruction"+) OR (MH "Data Collection"+) OR (MH "Data Mining"+) OR (MH "Educational Measurement"+) OR (MH "Multimethod Studies"+) OR (MH "Problem-Based Learning"+) OR (MH "Self-Directed Learning as Topic"+) OR (MH "Simulation Training"+)

S5 (((TI adaptive OR AB adaptive OR SU adaptive) OR (TI blended OR AB blended OR SU blended) OR (TI collaborative OR AB collaborative OR SU collaborative) OR (TI computer-based OR AB computer-based OR SU computer-based) OR (TI computer-mediated OR AB computer-mediated OR SU computer-mediated) OR (TI distance OR AB distance OR SU distance) OR (TI online OR AB online OR SU online) OR (TI personalized OR AB personalized OR SU personalized) OR (TI practical OR AB practical OR SU practical) OR (TI problem-based OR AB problem-based OR SU problem-based) OR (TI project-based OR AB project-based OR SU project-based) OR (TI self-directed OR AB self-directed OR SU self-directed) OR (TI self-regulated OR AB self-regulated OR SU self-regulated) OR (TI team-based OR AB team-based OR SU team-based) OR (TI technology-enhanced OR AB technology-enhanced OR SU technology-enhanced) OR (TI ubiquitous OR AB ubiquitous OR SU ubiquitous) OR (TI visual OR AB visual OR SU visual)) N2 ((TI learning OR AB learning OR SU learning) OR (TI education OR AB education OR SU education)))

S6 ((TI elearn* OR AB elearn* OR SU elearn*) OR (TI e-learn* OR AB e-learn* OR SU e-learn*) OR (TI "flipped classroom*" OR AB "flipped classroom*" OR SU "flipped classroom*") OR (TI mlearn* OR AB mlearn* OR SU mlearn*) OR (TI m-learn* OR AB m-learn* OR SU m-learn*) OR (TI "patient simulat*" OR AB "patient simulat*" OR SU "patient simulat*") OR (TI "virtual patient*" OR AB "virtual patient*" OR SU "virtual patient*") OR (TI "technology enhanced learning" OR AB "technology enhanced learning" OR SU "technology enhanced learning") OR (TI "massive open online course#" OR AB "massive open online course#" OR SU "massive open online course#"))

S7 ((TI Aquifer OR AB Aquifer OR SU Aquifer) OR (TI BioWord OR AB BioWord OR SU BioWord) OR (TI Blackboard OR AB Blackboard OR SU Blackboard) OR (TI "canvas network" OR AB "canvas network" OR SU "canvas network") OR (TI coursera OR AB coursera OR SU coursera) OR (TI coursesites OR AB coursesites OR SU coursesites) OR (TI edx OR AB edx OR SU edx) OR (TI e-MedEdu OR AB e-MedEdu OR SU e-MedEdu) OR (TI Equella OR AB Equella OR SU Equella) OR (TI futurelearn OR AB futurelearn OR SU futurelearn) OR (TI Hypocampus OR AB Hypocampus OR SU Hypocampus) OR (TI iversity OR AB iversity OR SU iversity) OR (TI Kaizen OR AB Kaizen OR SU Kaizen) OR (TI McPeer OR AB McPeer OR SU McPeer) OR (TI MedU OR AB MedU OR SU MedU) OR (TI "miriada x" OR AB "miriada x" OR SU "miriada x") OR (TI Moodle OR AB Moodle OR SU Moodle) OR (TI novoed OR AB novoed OR SU novoed) OR (TI open2study OR AB open2study OR SU open2study) OR (TI openlearning OR AB openlearning OR SU openlearning) OR (TI Osmosis OR AB Osmosis OR SU Osmosis) OR (TI pingpong OR AB pingpong OR SU pingpong) OR (TI plato OR AB plato OR SU plato) OR (TI "Smart Sparrow" OR AB "Smart Sparrow" OR SU "Smart Sparrow") OR (TI spoc OR AB spoc OR SU spoc) OR (TI udacity OR AB udacity OR SU udacity) OR (TI Vquest OR AB Vquest OR SU Vquest))

S8 (("technologically enhanced" OR computer-* OR "medical school" OR adaptive OR digital OR virtual OR online OR e- OR blended ) N2 (learning OR education* ) OR "online assessment" OR "intelligent tutoring" OR "smart tutoring" OR "e-learning management" OR "learning management" OR "learning content management" ) N2 ((TI dashboard* OR AB dashboard* OR SU dashboard*) OR (TI environment* OR AB environment* OR SU environment*) OR (TI platform* OR AB platform* OR SU platform*) OR (TI portal* OR AB portal* OR SU portal*) OR (TI software OR AB software OR SU software) OR (TI system* OR AB system* OR SU system*))

S9 ((TI "learning analytics" OR AB "learning analytics" OR SU "learning analytics") OR (TI "academic analytics" OR AB "academic analytics" OR SU "academic analytics") OR (TI "educational analytics" OR AB "educational analytics" OR SU "educational analytics") OR (TI "educational data mining" OR AB "educational data mining" OR SU "educational data mining"))

S10 (S1 OR S2 OR S3) AND (S4 OR S5 OR S6 OR S7 OR S8) AND S9

S11 "limit S10 to" ("english language" AND "yr="2010 - 2021"" )

# Cochrane

#1 [mh "biomedical engineering"] OR [mh chiropractic] OR [mh dentistry] OR [mh dietetics] OR [mh optometry] OR [mh orthoptics] OR [mh pharmacology] OR [mh pharmacy] OR [mh podiatry] OR [mh "psychology, medical"] OR [mh radiography] OR [mh serology] OR [mh anatomists] OR [mh "coroners and medical examiners"] OR [mh "dental staff"] OR [mh dentists] OR [mh specialization] OR [mh "surgical procedures, operative"] OR [mh "infection control practitioners"] OR [mh pharmacists] OR [mh physicians] OR [mh "education, predental"] OR [mh "education, premedical"] OR [mh "education, professional"] NOT [mh ^"education, veterinary"] OR [mh "evidence-based practice"] OR [mh "health educators"] OR [mh medicine] OR [mh nursing] OR [mh "allied health occupations"] OR [mh "health occupations"] OR [mh "allied health personnel"] OR [mh "health personnel"] OR [mh "medical laboratory personnel"] OR [mh "medical staff"] OR [mh nurses] OR [mh "nursing staff"] OR [mh "personnel, hospital"] OR [mh "students, health occupations"]

#2 (clinic*:ti,ab,kw OR dent*:ti,ab,kw OR dietetic*:ti,ab,kw OR dieti?ian*:ti,ab,kw OR doctor*:ti,ab,kw OR ("general" NEXT practioner*):ti,ab,kw OR health*:ti,ab,kw OR ("hospital" NEXT administrator*):ti,ab,kw OR laborator*:ti,ab,kw OR medic*:ti,ab,kw OR nurs*:ti,ab,kw OR occupational*:ti,ab,kw OR pharmacist*:ti,ab,kw OR ("physical" NEXT therap*):ti,ab,kw OR physician*:ti,ab,kw OR physiotherap*:ti,ab,kw OR predent*:ti,ab,kw OR premedic*:ti,ab,kw OR therap*:ti,ab,kw)

#3 (an?esthesi*:ti,ab,kw OR cardiolog*:ti,ab,kw OR dermatolog*:ti,ab,kw OR gyn?ecolog*:ti,ab,kw OR neurolog*:ti,ab,kw OR nutrition*:ti,ab,kw OR obstetric*:ti,ab,kw OR oncolog*:ti,ab,kw OR orthodont*:ti,ab,kw OR orthopti*:ti,ab,kw OR p?ediatric*:ti,ab,kw OR patholog*:ti,ab,kw OR podiatr*:ti,ab,kw OR psychiatr*:ti,ab,kw OR psycholog*:ti,ab,kw OR radiolog*:ti,ab,kw OR rheumatolog*:ti,ab,kw OR surg*:ti,ab,kw OR urolog*:ti,ab,kw OR midwi?e*:ti,ab,kw) NEAR/3 (clerk*:ti,ab,kw OR educat*:ti,ab,kw OR fellow*:ti,ab,kw OR graduate*:ti,ab,kw OR internship*:ti,ab,kw OR novice*:ti,ab,kw OR personnel:ti,ab,kw OR practitioner*:ti,ab,kw OR residen*:ti,ab,kw OR staff:ti,ab,kw OR student*:ti,ab,kw OR train*:ti,ab,kw OR tutor*:ti,ab,kw OR undergraduate*:ti,ab,kw)

#4 [mh "education, distance"] OR [mh "educational technology"] OR [mh "models, anatomic"] OR [mh "patient simulation"] OR [mh "Computer-Assisted Instruction"] OR [mh "Data Collection"] OR [mh "Data Mining"] OR [mh "Educational Measurement"] OR [mh "Multimethod Studies"] OR [mh "Problem-Based Learning"] OR [mh "Self-Directed Learning as Topic"] OR [mh "Simulation Training"]

#5 ((adaptive:ti,ab,kw OR blended:ti,ab,kw OR collaborative:ti,ab,kw OR computer-based:ti,ab,kw OR computer-mediated:ti,ab,kw OR distance:ti,ab,kw OR online:ti,ab,kw OR personalized:ti,ab,kw OR practical:ti,ab,kw OR problem-based:ti,ab,kw OR project-based:ti,ab,kw OR self-directed:ti,ab,kw OR self-regulated:ti,ab,kw OR team-based:ti,ab,kw OR technology-enhanced:ti,ab,kw OR ubiquitous:ti,ab,kw OR visual:ti,ab,kw) NEAR/2 (learning:ti,ab,kw OR education:ti,ab,kw))

#6 (elearn*:ti,ab,kw OR e-learn*:ti,ab,kw OR ("flipped" NEXT classroom*):ti,ab,kw OR mlearn*:ti,ab,kw OR m-learn*:ti,ab,kw OR ("patient" NEXT simulat*):ti,ab,kw OR ("virtual" NEXT patient*):ti,ab,kw OR "technology enhanced learning":ti,ab,kw OR ("massive open online" NEXT course?):ti,ab,kw)

#7 (Aquifer:ti,ab,kw OR BioWord:ti,ab,kw OR Blackboard:ti,ab,kw OR "canvas network":ti,ab,kw OR coursera:ti,ab,kw OR coursesites:ti,ab,kw OR edx:ti,ab,kw OR e-MedEdu:ti,ab,kw OR Equella:ti,ab,kw OR futurelearn:ti,ab,kw OR Hypocampus:ti,ab,kw OR iversity:ti,ab,kw OR Kaizen:ti,ab,kw OR McPeer:ti,ab,kw OR MedU:ti,ab,kw OR "miriada x":ti,ab,kw OR Moodle:ti,ab,kw OR novoed:ti,ab,kw OR open2study:ti,ab,kw OR openlearning:ti,ab,kw OR Osmosis:ti,ab,kw OR pingpong:ti,ab,kw OR plato:ti,ab,kw OR "Smart Sparrow":ti,ab,kw OR spoc:ti,ab,kw OR udacity:ti,ab,kw OR Vquest:ti,ab,kw)

#8 (("technologically enhanced" OR computer-* OR "medical school" OR adaptive OR digital OR virtual OR online OR e- OR blended ) NEAR/2 (learning OR education* ) OR "online assessment" OR "intelligent tutoring" OR "smart tutoring" OR "e-learning management" OR "learning management" OR "learning content management" ) NEAR/2 (dashboard*:ti,ab,kw OR environment*:ti,ab,kw OR platform*:ti,ab,kw OR portal*:ti,ab,kw OR software:ti,ab,kw OR system*:ti,ab,kw)

#9 ("learning analytics":ti,ab,kw OR "academic analytics":ti,ab,kw OR "educational analytics":ti,ab,kw OR "educational data mining":ti,ab,kw)

#10 (#1 OR #2 OR #3) AND (#4 OR #5 OR #6 OR #7 OR #8) AND #9

#11 "limit #10 to" ("english language" AND "yr="2010 - 2021"" )

# Scopus

#1 ((INDEXTERMS("biomedical engineering") OR INDEXTERMS(chiropractic) OR INDEXTERMS(dentistry) OR INDEXTERMS(dietetics) OR INDEXTERMS(optometry) OR INDEXTERMS(orthoptics) OR INDEXTERMS(pharmacology) OR INDEXTERMS(pharmacy) OR INDEXTERMS(podiatry) OR INDEXTERMS("psychology, medical") OR INDEXTERMS(radiography) OR INDEXTERMS(serology) OR INDEXTERMS(anatomists) OR INDEXTERMS("coroners and medical examiners") OR INDEXTERMS("dental staff") OR INDEXTERMS(dentists) OR INDEXTERMS(specialization) OR INDEXTERMS("surgical procedures, operative") OR INDEXTERMS("infection control practitioners") OR INDEXTERMS(pharmacists) OR INDEXTERMS(physicians) OR INDEXTERMS("education, predental") OR INDEXTERMS("education, premedical") OR INDEXTERMS("education, professional") NOT INDEXTERMS("education, veterinary") OR INDEXTERMS("evidence-based practice") OR INDEXTERMS("health educators") OR INDEXTERMS(medicine) OR INDEXTERMS(nursing) OR INDEXTERMS("allied health occupations") OR INDEXTERMS("health occupations") OR INDEXTERMS("allied health personnel") OR INDEXTERMS("health personnel") OR INDEXTERMS("medical laboratory personnel") OR INDEXTERMS("medical staff") OR INDEXTERMS(nurses) OR INDEXTERMS("nursing staff") OR INDEXTERMS("personnel, hospital") OR INDEXTERMS("students, health occupations"))

#2 TITLE-ABS-KEY(clinic* OR dent* OR dietetic* OR dieti*ian* OR doctor* OR "general practioner*" OR health* OR "hospital administrator*" OR laborator* OR medic* OR nurs* OR occupational* OR pharmacist* OR "physical therap*" OR physician* OR physiotherap* OR predent* OR premedic* OR therap* )

#3 (TITLE-ABS-KEY(an*esthesi* OR cardiolog* OR dermatolog* OR gyn*ecolog* OR neurolog* OR nutrition* OR obstetric* OR oncolog* OR orthodont* OR orthopti* OR p*ediatric* OR patholog* OR podiatr* OR psychiatr* OR psycholog* OR radiolog* OR rheumatolog* OR surg* OR urolog* OR midwi*e* ) W/3 TITLE-ABS-KEY(clerk* OR educat* OR fellow* OR graduate* OR internship* OR novice* OR personnel OR practitioner* OR residen* OR staff OR student* OR train* OR tutor* OR undergraduate* )))

#4 ((INDEXTERMS("education, distance") OR INDEXTERMS("educational technology") OR INDEXTERMS("models, anatomic") OR INDEXTERMS("patient simulation") OR INDEXTERMS("Computer-Assisted Instruction") OR INDEXTERMS("Data Collection") OR INDEXTERMS("Data Mining") OR INDEXTERMS("Educational Measurement") OR INDEXTERMS("Multimethod Studies") OR INDEXTERMS("Problem-Based Learning") OR INDEXTERMS("Self-Directed Learning as Topic") OR INDEXTERMS("Simulation Training"))

#5 TITLE-ABS-KEY((adaptive OR blended OR collaborative OR computer-based OR computer-mediated OR distance OR online OR personalized OR practical OR problem-based OR project-based OR self-directed OR self-regulated OR team-based OR technology-enhanced OR ubiquitous OR visual ) W/2 (learning OR education ))

#6 TITLE-ABS-KEY(elearn* OR e-learn* OR "flipped classroom*" OR mlearn* OR m-learn* OR "patient simulat*" OR "virtual patient*" OR "technology enhanced learning" OR "massive open online course*" )

#7 TITLE-ABS-KEY(("online assessment" OR "intelligent tutoring" OR "smart tutoring" OR "e-learning management" OR "learning management" OR "learning content management" OR ("technologically enhanced" OR computer-* OR "medical school" OR adaptive OR digital OR virtual OR online OR e- OR blended ) W/2 (learning OR education* )) W/2 (dashboard* OR environment* OR platform* OR portal* OR software OR system* ))

#8 TITLE-ABS-KEY(Aquifer OR BioWord OR Blackboard OR "canvas network" OR coursera OR coursesites OR edx OR e-MedEdu OR Equella OR futurelearn OR Hypocampus OR iversity OR Kaizen OR McPeer OR MedU OR "miriada x" OR Moodle OR novoed OR open2study OR openlearning OR Osmosis OR pingpong OR plato OR "Smart Sparrow" OR spoc OR udacity OR Vquest ))

#9 TITLE-ABS-KEY("learning analytics" OR "academic analytics" OR "educational analytics" OR "educational data mining" )

#10 (#1 OR #2 OR #3) AND (#4 OR #5 OR #6 OR #7 OR #8) AND #9

#11 "limit #10 to" ("english language" AND "yr="2010 - 2021"" )

# Web of Science

#1 (ALL="biomedical engineering" OR ALL=chiropractic OR ALL=dentistry OR ALL=dietetics OR ALL=optometry OR ALL=orthoptics OR ALL=pharmacology OR ALL=pharmacy OR ALL=podiatry OR ALL="psychology, medical" OR ALL=radiography OR ALL=serology OR ALL=anatomists OR ALL="coroners and medical examiners" OR ALL="dental staff" OR ALL=dentists OR ALL=specialization OR ALL="surgical procedures, operative" OR ALL="infection control practitioners" OR ALL=pharmacists OR ALL=physicians OR ALL="education, predental" OR ALL="education, premedical" OR ALL="education, professional" NOT ALL="education, veterinary" OR ALL="evidence-based practice" OR ALL="health educators" OR ALL=medicine OR ALL=nursing OR ALL="allied health occupations" OR ALL="health occupations" OR ALL="allied health personnel" OR ALL="health personnel" OR ALL="medical laboratory personnel" OR ALL="medical staff" OR ALL=nurses OR ALL="nursing staff" OR ALL="personnel, hospital" OR ALL="students, health occupations")

#2 TS=(clinic* OR dent* OR dietetic* OR dieti$ian* OR doctor* OR "general practioner*" OR health* OR "hospital administrator*" OR laborator* OR medic* OR nurs* OR occupational* OR pharmacist* OR "physical therap*" OR physician* OR physiotherap* OR predent* OR premedic* OR therap* )

#3 TS=(an$esthesi* NEAR/3 internship* OR cardiolog* NEAR/3 internship* OR dermatolog* NEAR/3 internship* OR gyn$ecolog* NEAR/3 internship* OR neurolog* NEAR/3 internship* OR nutrition* NEAR/3 internship* OR obstetric* NEAR/3 internship* OR oncolog* NEAR/3 internship* OR orthodont* NEAR/3 internship* OR orthopti* NEAR/3 internship* OR p$ediatric* NEAR/3 internship* OR patholog* NEAR/3 internship* OR podiatr* NEAR/3 internship* OR psychiatr* NEAR/3 internship* OR psycholog* NEAR/3 internship* OR radiolog* NEAR/3 internship* OR rheumatolog* NEAR/3 internship* OR surg* NEAR/3 internship* OR urolog* NEAR/3 internship* OR midwi$e* NEAR/3 internship*)

#4 TS=(an$esthesi* NEAR/3 novice* OR cardiolog* NEAR/3 novice* OR dermatolog* NEAR/3 novice* OR gyn$ecolog* NEAR/3 novice* OR neurolog* NEAR/3 novice* OR nutrition* NEAR/3 novice* OR obstetric* NEAR/3 novice* OR oncolog* NEAR/3 novice* OR orthodont* NEAR/3 novice* OR orthopti* NEAR/3 novice* OR p$ediatric* NEAR/3 novice* OR patholog* NEAR/3 novice* OR podiatr* NEAR/3 novice* OR psychiatr* NEAR/3 novice* OR psycholog* NEAR/3 novice* OR radiolog* NEAR/3 novice* OR rheumatolog* NEAR/3 novice* OR surg* NEAR/3 novice* OR urolog* NEAR/3 novice* OR midwi$e* NEAR/3 novice*)

#5 TS=(an$esthesi* NEAR/3 personnel OR cardiolog* NEAR/3 personnel OR dermatolog* NEAR/3 personnel OR gyn$ecolog* NEAR/3 personnel OR neurolog* NEAR/3 personnel OR nutrition* NEAR/3 personnel OR obstetric* NEAR/3 personnel OR oncolog* NEAR/3 personnel OR orthodont* NEAR/3 personnel OR orthopti* NEAR/3 personnel OR p$ediatric* NEAR/3 personnel OR patholog* NEAR/3 personnel OR podiatr* NEAR/3 personnel OR psychiatr* NEAR/3 personnel OR psycholog* NEAR/3 personnel OR radiolog* NEAR/3 personnel OR rheumatolog* NEAR/3 personnel OR surg* NEAR/3 personnel OR urolog* NEAR/3 personnel OR midwi$e* NEAR/3 personnel) OR

#6 TS=(an$esthesi* NEAR/3 practitioner* OR cardiolog* NEAR/3 practitioner* OR dermatolog* NEAR/3 practitioner* OR gyn$ecolog* NEAR/3 practitioner* OR neurolog* NEAR/3 practitioner* OR nutrition* NEAR/3 practitioner* OR obstetric* NEAR/3 practitioner* OR oncolog* NEAR/3 practitioner* OR orthodont* NEAR/3 practitioner* OR orthopti* NEAR/3 practitioner* OR p$ediatric* NEAR/3 practitioner* OR patholog* NEAR/3 practitioner* OR podiatr* NEAR/3 practitioner* OR psychiatr* NEAR/3 practitioner* OR psycholog* NEAR/3 practitioner* OR radiolog* NEAR/3 practitioner* OR rheumatolog* NEAR/3 practitioner* OR surg* NEAR/3 practitioner* OR urolog* NEAR/3 practitioner* OR midwi$e* NEAR/3 practitioner*) OR

#7 TS=(an$esthesi* NEAR/3 residen* OR cardiolog* NEAR/3 residen* OR dermatolog* NEAR/3 residen* OR gyn$ecolog* NEAR/3 residen* OR neurolog* NEAR/3 residen* OR nutrition* NEAR/3 residen* OR obstetric* NEAR/3 residen* OR oncolog* NEAR/3 residen* OR orthodont* NEAR/3 residen* OR orthopti* NEAR/3 residen* OR p$ediatric* NEAR/3 residen* OR patholog* NEAR/3 residen* OR podiatr* NEAR/3 residen* OR psychiatr* NEAR/3 residen* OR psycholog* NEAR/3 residen* OR radiolog* NEAR/3 residen* OR rheumatolog* NEAR/3 residen* OR surg* NEAR/3 residen* OR urolog* NEAR/3 residen* OR midwi$e* NEAR/3 residen*) OR

#8 TS=(an$esthesi* NEAR/3 staff OR cardiolog* NEAR/3 staff OR dermatolog* NEAR/3 staff OR gyn$ecolog* NEAR/3 staff OR neurolog* NEAR/3 staff OR nutrition* NEAR/3 staff OR obstetric* NEAR/3 staff OR oncolog* NEAR/3 staff OR orthodont* NEAR/3 staff OR orthopti* NEAR/3 staff OR p$ediatric* NEAR/3 staff OR patholog* NEAR/3 staff OR podiatr* NEAR/3 staff OR psychiatr* NEAR/3 staff OR psycholog* NEAR/3 staff OR radiolog* NEAR/3 staff OR rheumatolog* NEAR/3 staff OR surg* NEAR/3 staff OR urolog* NEAR/3 staff OR midwi$e* NEAR/3 staff) OR

#9 TS=(an$esthesi* NEAR/3 student* OR cardiolog* NEAR/3 student* OR dermatolog* NEAR/3 student* OR gyn$ecolog* NEAR/3 student* OR neurolog* NEAR/3 student* OR nutrition* NEAR/3 student* OR obstetric* NEAR/3 student* OR oncolog* NEAR/3 student* OR orthodont* NEAR/3 student* OR orthopti* NEAR/3 student* OR p$ediatric* NEAR/3 student* OR patholog* NEAR/3 student* OR podiatr* NEAR/3 student* OR psychiatr* NEAR/3 student* OR psycholog* NEAR/3 student* OR radiolog* NEAR/3 student* OR rheumatolog* NEAR/3 student* OR surg* NEAR/3 student* OR urolog* NEAR/3 student* OR midwi$e* NEAR/3 student*) OR

#10 TS=(an$esthesi* NEAR/3 train* OR cardiolog* NEAR/3 train* OR dermatolog* NEAR/3 train* OR gyn$ecolog* NEAR/3 train* OR neurolog* NEAR/3 train* OR nutrition* NEAR/3 train* OR obstetric* NEAR/3 train* OR oncolog* NEAR/3 train* OR orthodont* NEAR/3 train* OR orthopti* NEAR/3 train* OR p$ediatric* NEAR/3 train* OR patholog* NEAR/3 train* OR podiatr* NEAR/3 train* OR psychiatr* NEAR/3 train* OR psycholog* NEAR/3 train* OR radiolog* NEAR/3 train* OR rheumatolog* NEAR/3 train* OR surg* NEAR/3 train* OR urolog* NEAR/3 train* OR midwi$e* NEAR/3 train*) OR

#11 TS=(an$esthesi* NEAR/3 tutor* OR cardiolog* NEAR/3 tutor* OR dermatolog* NEAR/3 tutor* OR gyn$ecolog* NEAR/3 tutor* OR neurolog* NEAR/3 tutor* OR nutrition* NEAR/3 tutor* OR obstetric* NEAR/3 tutor* OR oncolog* NEAR/3 tutor* OR orthodont* NEAR/3 tutor* OR orthopti* NEAR/3 tutor* OR p$ediatric* NEAR/3 tutor* OR patholog* NEAR/3 tutor* OR podiatr* NEAR/3 tutor* OR psychiatr* NEAR/3 tutor* OR psycholog* NEAR/3 tutor* OR radiolog* NEAR/3 tutor* OR rheumatolog* NEAR/3 tutor* OR surg* NEAR/3 tutor* OR urolog* NEAR/3 tutor* OR midwi$e* NEAR/3 tutor*) OR

#12 TS=(an$esthesi* NEAR/3 undergraduate* OR cardiolog* NEAR/3 undergraduate* OR dermatolog* NEAR/3 undergraduate* OR gyn$ecolog* NEAR/3 undergraduate* OR neurolog* NEAR/3 undergraduate* OR nutrition* NEAR/3 undergraduate* OR obstetric* NEAR/3 undergraduate* OR oncolog* NEAR/3 undergraduate* OR orthodont* NEAR/3 undergraduate* OR orthopti* NEAR/3 undergraduate* OR p$ediatric* NEAR/3 undergraduate* OR patholog* NEAR/3 undergraduate* OR podiatr* NEAR/3 undergraduate* OR psychiatr* NEAR/3 undergraduate* OR psycholog* NEAR/3 undergraduate* OR radiolog* NEAR/3 undergraduate* OR rheumatolog* NEAR/3 undergraduate* OR surg* NEAR/3 undergraduate* OR urolog* NEAR/3 undergraduate* OR midwi$e* NEAR/3 undergraduate*) OR

#13 TS=(an$esthesi* NEAR/3 clerk* OR cardiolog* NEAR/3 clerk* OR dermatolog* NEAR/3 clerk* OR gyn$ecolog* NEAR/3 clerk* OR neurolog* NEAR/3 clerk* OR nutrition* NEAR/3 clerk* OR obstetric* NEAR/3 clerk* OR oncolog* NEAR/3 clerk* OR orthodont* NEAR/3 clerk* OR orthopti* NEAR/3 clerk* OR p$ediatric* NEAR/3 clerk* OR patholog* NEAR/3 clerk* OR podiatr* NEAR/3 clerk* OR psychiatr* NEAR/3 clerk* OR psycholog* NEAR/3 clerk* OR radiolog* NEAR/3 clerk* OR rheumatolog* NEAR/3 clerk* OR surg* NEAR/3 clerk* OR urolog* NEAR/3 clerk* OR midwi$e* NEAR/3 clerk*) OR

#14 TS=(an$esthesi* NEAR/3 educat* OR cardiolog* NEAR/3 educat* OR dermatolog* NEAR/3 educat* OR gyn$ecolog* NEAR/3 educat* OR neurolog* NEAR/3 educat* OR nutrition* NEAR/3 educat* OR obstetric* NEAR/3 educat* OR oncolog* NEAR/3 educat* OR orthodont* NEAR/3 educat* OR orthopti* NEAR/3 educat* OR p$ediatric* NEAR/3 educat* OR patholog* NEAR/3 educat* OR podiatr* NEAR/3 educat* OR psychiatr* NEAR/3 educat* OR psycholog* NEAR/3 educat* OR radiolog* NEAR/3 educat* OR rheumatolog* NEAR/3 educat* OR surg* NEAR/3 educat* OR urolog* NEAR/3 educat* OR midwi$e* NEAR/3 educat*) OR

#15 TS=(an$esthesi* NEAR/3 fellow* OR cardiolog* NEAR/3 fellow* OR dermatolog* NEAR/3 fellow* OR gyn$ecolog* NEAR/3 fellow* OR neurolog* NEAR/3 fellow* OR nutrition* NEAR/3 fellow* OR obstetric* NEAR/3 fellow* OR oncolog* NEAR/3 fellow* OR orthodont* NEAR/3 fellow* OR orthopti* NEAR/3 fellow* OR p$ediatric* NEAR/3 fellow* OR patholog* NEAR/3 fellow* OR podiatr* NEAR/3 fellow* OR psychiatr* NEAR/3 fellow* OR psycholog* NEAR/3 fellow* OR radiolog* NEAR/3 fellow* OR rheumatolog* NEAR/3 fellow* OR surg* NEAR/3 fellow* OR urolog* NEAR/3 fellow* OR midwi$e* NEAR/3 fellow*) OR

#16 TS=(an$esthesi* NEAR/3 graduate* OR cardiolog* NEAR/3 graduate* OR dermatolog* NEAR/3 graduate* OR gyn$ecolog* NEAR/3 graduate* OR neurolog* NEAR/3 graduate* OR nutrition* NEAR/3 graduate* OR obstetric* NEAR/3 graduate* OR oncolog* NEAR/3 graduate* OR orthodont* NEAR/3 graduate* OR orthopti* NEAR/3 graduate* OR p$ediatric* NEAR/3 graduate* OR patholog* NEAR/3 graduate* OR podiatr* NEAR/3 graduate* OR psychiatr* NEAR/3 graduate* OR psycholog* NEAR/3 graduate* OR radiolog* NEAR/3 graduate* OR rheumatolog* NEAR/3 graduate* OR surg* NEAR/3 graduate* OR urolog* NEAR/3 graduate* OR midwi$e* NEAR/3 graduate*)

#17 (ALL="education, distance" OR ALL="educational technology" OR ALL="models, anatomic" OR ALL="patient simulation" OR ALL="Computer-Assisted Instruction" OR ALL="Data Collection" OR ALL="Data Mining" OR ALL="Educational Measurement" OR ALL="Multimethod Studies" OR ALL="Problem-Based Learning" OR ALL="Self-Directed Learning as Topic" OR ALL="Simulation Training")

#18 TS=(adaptive NEAR/2 learning OR blended NEAR/2 learning OR collaborative NEAR/2 learning OR computer-based NEAR/2 learning OR computer-mediated NEAR/2 learning OR distance NEAR/2 learning OR online NEAR/2 learning OR personalized NEAR/2 learning OR practical NEAR/2 learning OR problem-based NEAR/2 learning OR project-based NEAR/2 learning OR self-directed NEAR/2 learning OR self-regulated NEAR/2 learning OR team-based NEAR/2 learning OR technology-enhanced NEAR/2 learning OR ubiquitous NEAR/2 learning OR visual NEAR/2 learning)

#19 TS=(adaptive NEAR/2 education OR blended NEAR/2 education OR collaborative NEAR/2 education OR computer-based NEAR/2 education OR computer-mediated NEAR/2 education OR distance NEAR/2 education OR online NEAR/2 education OR personalized NEAR/2 education OR practical NEAR/2 education OR problem-based NEAR/2 education OR project-based NEAR/2 education OR self-directed NEAR/2 education OR self-regulated NEAR/2 education OR team-based NEAR/2 education OR technology-enhanced NEAR/2 education OR ubiquitous NEAR/2 education OR visual NEAR/2 learning)

#20 TS=(elearn* OR e-learn* OR "flipped classroom*" OR mlearn* OR m-learn* OR "patient simulat*" OR "virtual patient*" OR "technology enhanced learning" OR "massive open online course$" )

#21 TS=("online assessment" NEAR/2 software OR "intelligent tutoring" NEAR/2 software OR "smart tutoring" NEAR/2 software OR "e-learning management" NEAR/2 software OR "learning management" NEAR/2 software OR "learning content management" NEAR/2 software OR "technologically enhanced” learning NEAR/2 software OR computer-* learning NEAR/2 software OR "medical school" learning NEAR/2 software OR adaptive learning NEAR/2 software OR digital learning NEAR/2 software OR virtual learning NEAR/2 software OR online learning NEAR/2 software OR e- learning NEAR/2 software OR blended learning NEAR/2 software OR "technologically enhanced" education* NEAR/2 software OR computer-* education* NEAR/2 software OR "medical school" education* NEAR/2 software OR adaptive education* NEAR/2 software OR digital education* NEAR/2 software OR virtual education* NEAR/2 software OR online education* NEAR/2 software OR e- education* NEAR/2 software OR blended education*)

#22 TS=("online assessment" NEAR/2 system* OR "intelligent tutoring" NEAR/2 system* OR "smart tutoring" NEAR/2 system* OR "e-learning management" NEAR/2 system* OR "learning management" NEAR/2 system* OR "learning content management" NEAR/2 system* OR "technologically enhanced” learning NEAR/2 system* OR computer-* learning NEAR/2 system* OR "medical school" learning NEAR/2 system* OR adaptive learning NEAR/2 system* OR digital learning NEAR/2 system* OR virtual learning NEAR/2 system* OR online learning NEAR/2 system* OR e- learning NEAR/2 system* OR blended learning NEAR/2 system* OR "technologically enhanced" education* NEAR/2 system* OR computer-* education* NEAR/2 system* OR "medical school" education* NEAR/2 system* OR adaptive education* NEAR/2 system* OR digital education* NEAR/2 system* OR virtual education* NEAR/2 system* OR online education* NEAR/2 system* OR e- education* NEAR/2 system* OR blended education*)

#23 TS=("online assessment" NEAR/2 portal* OR "intelligent tutoring" NEAR/2 portal* OR "smart tutoring" NEAR/2 portal* OR "e-learning management" NEAR/2 portal* OR "learning management" NEAR/2 portal* OR "learning content management" NEAR/2 portal* OR "technologically enhanced” learning NEAR/2 portal* OR computer-* learning NEAR/2 portal* OR "medical school" learning NEAR/2 portal* OR adaptive learning NEAR/2 portal* OR digital learning NEAR/2 portal* OR virtual learning NEAR/2 portal* OR online learning NEAR/2 portal* OR e- learning NEAR/2 portal* OR blended learning NEAR/2 portal* OR "technologically enhanced" education* NEAR/2 portal* OR computer-* education* NEAR/2 portal* OR "medical school" education* NEAR/2 portal* OR adaptive education* NEAR/2 portal* OR digital education* NEAR/2 portal* OR virtual education* NEAR/2 portal* OR online education* NEAR/2 portal* OR e- education* NEAR/2 portal* OR blended education*)

#24 TS=("online assessment" NEAR/2 platform* OR "intelligent tutoring" NEAR/2 platform* OR "smart tutoring" NEAR/2 platform* OR "e-learning management" NEAR/2 platform* OR "learning management" NEAR/2 platform* OR "learning content management" NEAR/2 platform* OR "technologically enhanced” learning NEAR/2 platform* OR computer-* learning NEAR/2 platform* OR "medical school" learning NEAR/2 platform* OR adaptive learning NEAR/2 platform* OR digital learning NEAR/2 platform* OR virtual learning NEAR/2 platform* OR online learning NEAR/2 platform* OR e- learning NEAR/2 platform* OR blended learning NEAR/2 platform* OR "technologically enhanced" education* NEAR/2 platform* OR computer-* education* NEAR/2 platform* OR "medical school" education* NEAR/2 platform* OR adaptive education* NEAR/2 platform* OR digital education* NEAR/2 platform* OR virtual education* NEAR/2 platform* OR online education* NEAR/2 platform* OR e- education* NEAR/2 platform* OR blended education*)

#25 TS=("online assessment" NEAR/2 environment* OR "intelligent tutoring" NEAR/2 environment* OR "smart tutoring" NEAR/2 environment* OR "e-learning management" NEAR/2 environment* OR "learning management" NEAR/2 environment* OR "learning content management" NEAR/2 environment* OR "technologically enhanced” learning NEAR/2 environment* OR computer-* learning NEAR/2 environment* OR "medical school" learning NEAR/2 environment* OR adaptive learning NEAR/2 environment* OR digital learning NEAR/2 environment* OR virtual learning NEAR/2 environment* OR online learning NEAR/2 environment* OR e- learning NEAR/2 environment* OR blended learning NEAR/2 environment* OR "technologically enhanced" education* NEAR/2 environment* OR computer-* education* NEAR/2 environment* OR "medical school" education* NEAR/2 environment* OR adaptive education* NEAR/2 environment* OR digital education* NEAR/2 environment* OR virtual education* NEAR/2 environment* OR online education* NEAR/2 environment* OR e- education* NEAR/2 environment* OR blended education*)

#26 TS=("online assessment" NEAR/2 dashboard* OR "intelligent tutoring" NEAR/2 dashboard* OR "smart tutoring" NEAR/2 dashboard* OR "e-learning management" NEAR/2 dashboard* OR "learning management" NEAR/2 dashboard* OR "learning content management" NEAR/2 dashboard* OR "technologically enhanced” learning NEAR/2 dashboard* OR computer-* learning NEAR/2 dashboard* OR "medical school" learning NEAR/2 dashboard* OR adaptive learning NEAR/2 dashboard* OR digital learning NEAR/2 dashboard* OR virtual learning NEAR/2 dashboard* OR online learning NEAR/2 dashboard* OR e- learning NEAR/2 dashboard* OR blended learning NEAR/2 dashboard* OR "technologically enhanced" education* NEAR/2 dashboard* OR computer-* education* NEAR/2 dashboard* OR "medical school" education* NEAR/2 dashboard* OR adaptive education* NEAR/2 dashboard* OR digital education* NEAR/2 dashboard* OR virtual education* NEAR/2 dashboard* OR online education* NEAR/2 dashboard* OR e- education* NEAR/2 dashboard* OR blended education*)

#27 TS=(Aquifer OR BioWord OR Blackboard OR "canvas network" OR coursera OR coursesites OR edx OR e-MedEdu OR Equella OR futurelearn OR Hypocampus OR iversity OR Kaizen OR McPeer OR MedU OR "miriada x" OR Moodle OR novoed OR open2study OR openlearning OR Osmosis OR pingpong OR plato OR "Smart Sparrow" OR spoc OR udacity OR Vquest ))

#28 TS=("learning analytics" OR "academic analytics" OR "educational analytics" OR "educational data mining" )

#29 (#1 OR #2 OR #3 OR #4 OR #5 OR #6 OR #7 OR #8 OR #9 OR #10 OR #11 OR #12 OR #13 OR #14 OR #15 OR #16) AND (#17 OR #18 #19 OR #20 OR #21 OR #22 OR #23 OR #24 OR #25 OR #26 OR #27) AND #28

#30 "limit #29 to" ("english language" AND "yr="2010 - 2021"" )

# IEEE Explore

(

(“Mesh_Terms”: biomedical engineering OR “Mesh_Terms”: chiropractic OR “Mesh_Terms”: dentistry OR “Mesh_Terms”: dietetics OR “Mesh_Terms”: optometry OR “Mesh_Terms”: orthoptics OR “Mesh_Terms”: pharmacology OR “Mesh_Terms”: pharmacy OR “Mesh_Terms”: podiatry OR “Mesh_Terms”: psychology, medical OR “Mesh_Terms”: radiography OR “Mesh_Terms”: serology OR “Mesh_Terms”: anatomists OR “Mesh_Terms”: coroners and medical examiners OR “Mesh_Terms”: dental staff OR “Mesh_Terms”: dentists OR “Mesh_Terms”: specialization OR “Mesh_Terms”: surgical procedures, operative OR “Mesh_Terms”: infection control practitioners OR “Mesh_Terms”: pharmacists OR “Mesh_Terms”: physicians OR “Mesh_Terms”: education, predental OR “Mesh_Terms”: education, premedical OR “Mesh_Terms”: education, professional/ not education, veterinary OR “Mesh_Terms”: evidence-based practice OR “Mesh_Terms”: health educators OR “Mesh_Terms”: medicine OR “Mesh_Terms”: nursing OR “Mesh_Terms”: allied health occupations OR “Mesh_Terms”: health occupations OR “Mesh_Terms”: allied health personnel OR “Mesh_Terms”: health personnel OR “Mesh_Terms”: medical laboratory personnel OR “Mesh_Terms”: medical staff OR “Mesh_Terms”: nurses OR “Mesh_Terms”: nursing staff OR “Mesh_Terms”: personnel, hospital OR “Mesh_Terms”: students, health occupations)

**OR**

(“All Metadata”: clinician OR “All Metadata”: clinicians OR “All Metadata”: clinical OR “All Metadata”: dentist OR “All Metadata”: dentists OR “All Metadata”: dental OR “All Metadata”: Dietetician OR “All Metadata”: Dieteticians OR “All Metadata”: dietitian OR “All Metadata”: dietitians OR “All Metadata”: dietician OR “All Metadata”: dieticians OR “All Metadata”: Doctor OR “All Metadata”: Doctors OR “All Metadata”: General practitioner OR “All Metadata”: General practitioners OR “All Metadata”: health OR “All Metadata”: healthcare OR “All Metadata”: Hospital Administrators OR “All Metadata”: laboratorian OR “All Metadata”: laboratorians OR “All Metadata”: medic OR “All Metadata”: medics OR “All Metadata”: Nurse OR “All Metadata”: Nurses OR “All Metadata”: occupational therapist OR “All Metadata”: occupational therapists OR “All Metadata”: Pharmacist OR “All Metadata”: Pharmacists OR “All Metadata”: Physical therapist OR “All Metadata”: Physical therapists OR “All Metadata”: Physician OR “All Metadata”: Physicians OR “All Metadata”: Physiotherapist OR “All Metadata”: Physiotherapists OR “All Metadata”: predental OR “All Metadata”: predentist OR “All Metadata”: premedical OR “All Metadata”: premedicals OR “All Metadata”: therapist OR “All Metadata”: therapists)

**OR**

(

(“All Metadata”: Anesthetist OR “All Metadata”: Anesthetists OR “All Metadata”: Anaesthetist OR “All Metadata”: Anaesthetists OR “All Metadata”: Cardiologist OR “All Metadata”: Cardiologists OR “All Metadata”: Dermatologist OR “All Metadata”: Dermatologists OR “All Metadata”: gynecologist OR “All Metadata”: gynecologists OR “All Metadata”: gynaecologist OR “All Metadata”: gynaecologists OR “All Metadata”: Neurologist OR “All Metadata”: Neurologists OR “All Metadata”: nutritionist OR “All Metadata”: nutritionists OR “All Metadata”: obstetrician OR “All Metadata”: obstetricians OR “All Metadata”: Oncologists OR “All Metadata”: Oncologist OR “All Metadata”: orthodontist OR “All Metadata”: orthodontists OR “All Metadata”: orthoptist OR “All Metadata”: orthoptists OR “All Metadata”: Pediatrician OR “All Metadata”: Pediatricians OR “All Metadata”: Paediatrician OR “All Metadata”: Paediatricians OR “All Metadata”: Pathologist OR “All Metadata”: Pathologists OR “All Metadata”: Podiatrist OR “All Metadata”: Podiatrists OR “All Metadata”: Psychiatrists OR “All Metadata”: Psychiatrist OR “All Metadata”: Psychologist OR “All Metadata”: Psychologists OR “All Metadata”: radiologist OR “All Metadata”: radiologists OR “All Metadata”: Rheumatologist OR “All Metadata”: Rheumatologists OR “All Metadata”: Surgeon OR “All Metadata”: Surgeons OR “All Metadata”: Urologist OR “All Metadata”: Urologists OR “All Metadata”: midwife OR “All Metadata”: midwives) NEAR/3 (“All Metadata”: clerk OR “All Metadata”: clerks OR “All Metadata”: educator OR “All Metadata”: educators OR “All Metadata”: fellow OR “All Metadata”: fellows OR “All Metadata”: graduate OR “All Metadata”: graduates OR “All Metadata”: internship OR “All Metadata”: internships OR “All Metadata”: novice OR “All Metadata”: novices OR “All Metadata”: personnel OR “All Metadata”: personnels OR “All Metadata”: practitioner OR “All Metadata”: practitioners OR “All Metadata”: resident OR “All Metadata”: residents OR “All Metadata”: staff OR “All Metadata”: student OR “All Metadata”: students OR “All Metadata”: trainers OR “All Metadata”: trainer OR “All Metadata”: tutor OR “All Metadata”: tutors OR “All Metadata”: undergraduate OR “All Metadata”: undergraduates)

)

)

**AND**

(

(“Mesh_Terms”: education, distance OR “Mesh_Terms”: educational technology OR “Mesh_Terms”: models, anatomic OR “Mesh_Terms”: patient simulation OR “Mesh_Terms”: Computer-Assisted Instruction OR “Mesh_Terms”: Data Collection OR “Mesh_Terms”: Data Mining OR “Mesh_Terms”: Educational Measurement OR “Mesh_Terms”: Multimethod Studies OR “Mesh_Terms”: Problem-Based Learning OR “Mesh_Terms”: Self-Directed Learning as Topic OR “Mesh_Terms”: Simulation Training)

**OR**

(

(“All Metadata”: adaptive OR “All Metadata”: blended OR “All Metadata”: collaborative OR “All Metadata”: computer-based OR “All Metadata”: computer-mediated OR “All Metadata”: distance OR “All Metadata”: online OR “All Metadata”: personalized OR “All Metadata”: practical OR “All Metadata”: problem-based OR “All Metadata”: project-based OR “All Metadata”: self-directed OR “All Metadata”: self-regulated OR “All Metadata”: team-based OR “All Metadata”: technology-enhanced OR “All Metadata”: ubiquitous OR “All Metadata”: visual) NEAR/2 (“All Metadata”: learning OR “All Metadata”: education)

)

**OR**

(“All Metadata”: elearn* OR “All Metadata”: e-learn* OR “All Metadata”: flipped classroom* OR “All Metadata”: mlearn* OR “All Metadata”: m-learn* OR “All Metadata”: patient simulation OR “All Metadata”: patient simulations OR “All Metadata”: virtual patient OR “All Metadata”: virtual patients OR “All Metadata”: technology enhanced learning OR “All Metadata”: massive open online course?)

**OR**

(

(“All Metadata”: adaptive OR “All Metadata”: “All Metadata”: blended OR “All Metadata”: “All Metadata”: collaborative OR “All Metadata”: “All Metadata”: computer-based OR “All Metadata”: “All Metadata”: computer-mediated OR “All Metadata”: “All Metadata”: distance OR “All Metadata”: “All Metadata”: online OR “All Metadata”: “All Metadata”: personalized OR “All Metadata”: “All Metadata”: practical OR “All Metadata”: “All Metadata”: problem-based OR “All Metadata”: “All Metadata”: project-based OR “All Metadata”: “All Metadata”: self-directed OR “All Metadata”: “All Metadata”: self-regulated OR “All Metadata”: “All Metadata”: team-based OR “All Metadata”: “All Metadata”: technology-enhanced OR “All Metadata”: “All Metadata”: ubiquitous OR “All Metadata”: “All Metadata”: visual) NEAR/2 (“All Metadata”: learning OR “All Metadata”: “All Metadata”: education)

)

**OR**

(“All Metadata”: Aquifer OR “All Metadata”: BioWord OR “All Metadata”: Blackboard OR “All Metadata”: canvas network OR “All Metadata”: coursera OR “All Metadata”: coursesites OR “All Metadata”: edx OR “All Metadata”: e-MedEdu OR “All Metadata”: Equella OR “All Metadata”: futurelearn OR “All Metadata”: Hypocampus OR “All Metadata”: iversity OR “All Metadata”: Kaizen OR “All Metadata”: McPeer OR “All Metadata”: MedU OR “All Metadata”: miriada x OR “All Metadata”: Moodle OR “All Metadata”: novoed OR “All Metadata”: open2study OR “All Metadata”: openlearning OR “All Metadata”: Osmosis OR “All Metadata”: pingpong OR “All Metadata”: plato OR “All Metadata”: Smart Sparrow OR “All Metadata”: spoc OR “All Metadata”: udacity OR “All Metadata”: Vquest)

)

**AND**

(“All Metadata”: learning analytics OR “All Metadata”: academic analytics OR “All Metadata”: educational analytics OR “All Metadata”: educational data mining)
